# CHASE-Containing Histidine Kinase Receptors in Apple Tree: From a Common Receptor Structure to Divergent Cytokinin Binding Properties and Specific Functions

**DOI:** 10.3389/fpls.2017.01614

**Published:** 2017-09-20

**Authors:** Dimitri Daudu, Elsa Allion, Franziska Liesecke, Nicolas Papon, Vincent Courdavault, Thomas Dugé de Bernonville, Céline Mélin, Audrey Oudin, Marc Clastre, Arnaud Lanoue, Martine Courtois, Olivier Pichon, David Giron, Sabine Carpin, Nathalie Giglioli-Guivarc’h, Joël Crèche, Sébastien Besseau, Gaëlle Glévarec

**Affiliations:** ^1^EA 2106 Biomolécules et Biotechnologies Végétales, Université François-Rabelais Tours, France; ^2^EA 3142 Groupe d’Etude des Interactions Hôte-Pathogène, Université Angers Angers, France; ^3^UMR 7261 Institut de Recherche sur la Biologie de l’Insecte, Centre National de la Recherche Scientifique (CNRS), Université François-Rabelais Tours, France; ^4^EA 1207 Laboratoire de Biologie des Ligneux et des Grandes Cultures, Université d’Orléans Orléans, France

**Keywords:** CHASE-containing histidine kinase, cytokinin, yeast complementation assay, RNAseq data, protein–protein interaction, *Malus domestica*

## Abstract

Cytokinin signaling is a key regulatory pathway of many aspects in plant development and environmental stresses. Herein, we initiated the identification and functional characterization of the five CHASE-containing histidine kinases (CHK) in the economically important *Malus domestica* species. These cytokinin receptors named MdCHK2, MdCHK3a/MdCHK3b, and MdCHK4a/MdCHK4b by homology with *Arabidopsis* AHK clearly displayed three distinct profiles. The three groups exhibited architectural variations, especially in the N-terminal part including the cytokinin sensing domain. Using a yeast complementation assay, we showed that MdCHK2 perceives a broad spectrum of cytokinins with a substantial sensitivity whereas both MdCHK4 homologs exhibit a narrow spectrum. Both MdCHK3 homologs perceived some cytokinins but surprisingly they exhibited a basal constitutive activity. Interaction studies revealed that MdCHK2, MdCHK4a, and MdCHK4b homodimerized whereas MdCHK3a and MdCHK3b did not. Finally, qPCR analysis and bioinformatics approach pointed out contrasted expression patterns among the three MdCHK groups as well as distinct sets of co-expressed genes. Our study characterized for the first time the five cytokinin receptors in apple tree and provided a framework for their further functional studies.

## Introduction

Cytokinins are essential adenine-derived plant hormones, gathering more than 40 structures substituted at the N^6^-position by an isoprenoid or aromatic chain (Spíchal, 2012; Osugi and Sakakibara, 2015). They are involved in numerous physiological processes such as cell division, delayed senescence, vascular tissue development, root architecture and light responses (Sakakibara, 2006; Kieber and Schaller, 2014; Zürcher and Müller, 2016). Cytokinins also play roles in the interaction with both biotic and abiotic factors (Frugier et al., 2008; Giron and Glévarec, 2014; Naseem et al., 2014; Zwack and Rashotte, 2015).

Plant cytokinin perception is mediated by CHASE domain-containing histidine kinase receptors (CHK) as first actors of cytokinin signaling (Inoue et al., 2001). These receptors display a complex multidomain structure with a N-terminal part including at least two hydrophobic membrane-spanning domains (TM) that border an extracytosolic sensing domain referred to as CHASE (Cyclase/Histidine kinase Associated Sensory Extracellular) (Anantharaman and Aravind, 2001; Mougel and Zhulin, 2001) as well as a cytoplasmic C-terminal part containing a catalytic histidine kinase domain (HK) and both receiver and pseudo-receiver domains (REC and REC-like, respectively) (Ueguchi et al., 2001). The HK domain is composed of an HK dimerization and phosphoacceptor domain (HisKA) and an HK catalytic domain called the HK-like ATPase domain (HATPase). The cytokinin perception by the CHASE domain leads to the autophosphorylation of a conserved histidine within the HK domain. The phosphate residue is then transferred to the REC domain on a conserved aspartate residue (Inoue et al., 2001). Although the pseudo-receiver domain of CHK is structurally similar to the REC domain, its functionality has not been yet elucidated (Ueguchi et al., 2001; Lomin et al., 2012). Subsequently, the signal is transferred by phosphorelay to Response Regulators (RR) through histidine-containing phosphotransfer shuttle proteins (HPt). While type-B RRs (RRB) are transcription factors that play a positive role in mediating cytokinin-regulated gene expression, type-A RRs (RRA) act as negative regulators of cytokinin responses (To et al., 2004; Mason et al., 2005; Ginis et al., 2012). In addition, Cytokinin Response Factors (CRF) interact directly with HPts and were reported to influence a subset of cytokinin responses (Cutcliffe et al., 2011; Raines et al., 2016).

Cytokinin receptors were shown to localize mainly to the endoplasmic reticulum (ER) both in *Arabidopsis thaliana* and *Zea mays* (Caesar et al., 2011; Lomin et al., 2011; Wulfetange et al., 2011). They are supposed to interact with each other, forming potential homo- and hetero-dimers probably enabling the *trans*-phosphorylation of the HK domain following cytokinin perception (Dortay et al., 2006; Hothorn et al., 2011). However, the signal transmission process across the membrane remains unknown. The ligand-binding properties of the cytokinin receptors have been investigated mostly using heterologous assay systems through their expression in *Escherichia coli* or *Saccharomyces cerevisiae* cells (Inoue et al., 2001; Romanov et al., 2006; Stolz et al., 2011; von Schwartzenberg et al., 2016). More recently, a plant assay system has been developed to overcome the problem of alien membrane environment and the difficulty to express some membrane receptors in bacteria or yeast (Lomin et al., 2015). Overall, the cytokinin receptors differ in their preference toward cytokinin forms (Yonekura-Sakakibara et al., 2004; Lomin et al., 2011; Kuderová et al., 2015) but their functional and specific properties as well as the structural changes caused by cytokinin binding remain to be elucidated.

While it is established that the CHK receptors operate mostly in a redundant fashion, the extensive studies of *Arabidopsis* mutants have attributed some specific roles to single receptors. Among others, AHK4 is the main regulator of primary root growth and vascular morphogenesis whereas AHK2 and AHK3 are commonly involved in chlorophyll retention during leaf senescence (Kim et al., 2006; Riefler et al., 2006). CHKs are also involved in response to environmental changes (Zwack and Rashotte, 2015). The three AHKs are also known to function as negative regulators in osmotic stress responses (Tran et al., 2007; Kumar and Verslues, 2015). AHK2 and AHK3 play an additional negative regulatory role in cold stress (Jeon et al., 2016) and ensure a protective function during light stress (Cortleven et al., 2014). Moreover, cytokinin receptors also take part in a large range of responses to biotic interactions. In legume plants, cytokinin receptors regulate nodule formation (Tirichine et al., 2007; Held et al., 2014; Boivin et al., 2016). In *Arabidopsis*, the success of the pathogens *Rhodococcus fascians* and *Hyaloperonospora arabidopsidis* depends on some AHKs (Pertry et al., 2009; Argueso et al., 2012). Finally, NaCHK2 and NaCHK3 modulate herbivory-induced defense signaling and defenses in *Nicotiana attenuata* (Schäfer et al., 2015). If the knowledge on the cytokinin receptors is increasingly important, their study in various plant models is necessary for a complete understanding of their biological functions.

Previous works on *M. domestica* reported a large accumulation of cytokinins in the leaves infected by the insect *Phyllonorycter blancardella*. This increase is responsible for the preservation of nutrient green tissues when leaves are otherwise turning yellow (Giron et al., 2007; Kaiser et al., 2010; Zhang et al., 2016). Based on the involvement of cytokinins in this plant-biotic interaction, we initiated the study of cytokinin signaling in apple tree with a special focus on cytokinin receptors. Indeed, considering that apple tree is one of the most cultivated fruit-tree with a continual worldwide production increase, a greater knowledge of cytokinin signaling pathway in this species could provide new opportunities for agronomical and economical purposes. This study discloses an overall and complete characterization of the five *M. domestica* CHASE Histidine Kinases (MdCHKs).

## Materials and Methods

### *In Silico* Sequence Analysis and Receptor Identification

To identify MdCHK receptors, BLAST searches were performed against the Genome Database for Rosaceae (GDR; Jung et al., 2014) using *A. thaliana* cytokinin receptor sequences as queries (AHK2, AHK3, and AHK4). Five sequences were identified based on genome analysis and corresponding cDNA were amplified from various plant organs using specific primers (Supplementary Table S1). Sequences were registered in Genbank as *MdCHK2* (KM114879), *MdCHK3a* (KM114880), *MdCHK3b* (KM114881), *MdCHK4a* (KM114883) and *MdCHK4b* (KM114882).

*MdCHKs* gene organization has been visualized using the FancyGene program (Rambaldi and Ciccarelli, 2009). Phylogeny analyses were performed on conserved domains and local similarities among proteins sequences. To this aim, multiple protein sequence alignments were done using the COBALT tool (Papadopoulos and Agarwala, 2007) and sequences were curated with Gblocks prior the construction of a bootstrap neighbor joining tree. Protein domain predictions were acquired using the SMART (Letunic et al., 2015) and PROSITE (Sigrist et al., 2002) programs, and transmembrane regions were identified with TMHMM (Krogh et al., 2001) and TMpred tools (Hofmann and Stoffel, 1993). Visualization of the transmembrane helixes has been performed with a helical wheel drawing program^1^.

### Yeast Complementation Assay

The *S. cerevisiae* strain YIL147C, deficient in SLN1 receptor (*MATa*/α, *ura3, leu2, his3, can1*Δ::*LEU2-MFA1*pro-*HIS3/CAN1, sln1*Δ::*KanMX*/*SLN1*) was used in complementation assays. Full-length coding sequences of MdCHKs were amplified and cloned into the yeast expression vector pYES2 under the control of the *GAL1*gene promoter using the NotI restriction site (for primers, see Supplementary Table S1). The *S. Cerevisiae* strain was transformed as follows. Cells were grown in 200 mL YPD liquid medium (150 rpm, 28°C) to 0.4–0.6 OD_600_, harvested by centrifugation (3000 *g*, 10 min) and resuspended in 0.1 M lithium acetate, 10 mMTris–HCl, pH 7.5,1 mM EDTA, 10 mM DTT. After 1 h incubation at 30°C, cells were washed twice with ice-cold 1 M sorbitol and resuspended in 1–5 mL ice-cold 1 M sorbitol. Plasmid DNA (0.5–1 μg DNA) was added to a 180 μL cell suspension and transferred to a 0.2 cm gap width electroporation cuvette. Electroporation was performed using a Bio-Rad gene pulser with an electric pulse of 2.5 kV, 25 μF and 200 Ω. Cells were immediately washed out from the cuvettes after the electroporation with YPD, plated on selective CSM-URA medium and incubated at 28°C for 3–5 days. Fresh colonies were then grown 3 h in liquid YPD at 30°C and meiosis was induced by pouring the suspension cell on ACK medium (10 g/L potassium acetate, 2.5 g/L yeast extract, 20 g/L agar). These plates were incubated 1 week at 20°C and haploids were finally selected on MMAS medium plates [20 g/L galactose, 7 g/L YNB (WA), 0.6 g/L DOB-LEU-HIS-ARG-URA, 0.06 g/L L-canavanine, 0.2 g/L G418, 20 g/L agar] supplemented with 10 μM *trans*-zeatin at 28°C for 3–5 days. Suspensions of transformants were then spotted onto dropout media containing or not 10 μM of *trans*-zeatin with 2% galactose and grown for 48 h at 28°C. For specificity and sensitivity assays, complemented-yeast growth was carried out in liquid YCGal (7 g/L YNB, 0.8 g/L CSM-URA, 20 g/L galactose) supplemented with various types and concentrations of cytokinins for 48 h at 28°C. Cell growth was measured at 630 nm (BioHit Reader BP800).

### Chemicals

Pure standards of isopentenyladenosine 5′-monophosphate, *trans*-zeatin riboside 5′-monophosphate, *cis*-zeatin riboside 5′- monophosphate, isopentenyladenine, *trans*-zeatin, *cis*-zeatin, dihydrozeatin, isopentenyladenosine, *trans*-zeatin riboside, *cis*-zeatin riboside, dihydrozeatin riboside, dihydrozeatin riboside 5′-monophosphate, *trans*-zeatin *O*-glucoside, *trans*-zeatin O-glucoside riboside, *trans*-zeatin N7-glucoside, 2- methylthio-isopentyladenine, 2-methylthio-*trans*-zeatin, 2-methylthio-*cis*-zeatin, 2-methylthio-isopentyladenosine, 2-methylthio-*trans*-zeatin riboside, 2-methylthio-*cis*-zeatin riboside were purchased from Olchemim (Olomouc, Czechia).

### RNA Isolation and Gene Expression Analysis

Extraction of total RNA from *M. domestica* organs was performed using the NucleoSpin RNA extraction kit (Marcherey-Nagel), with improved lysis step (McKenzie et al., 1997). First-strand cDNA were synthesized from 1 μg of total RNA using the iScript cDNA Synthesis Kit (Bio-Rad). Quantitative real-time PCR measurements were carried out in triplicate using SsoAdvanced Universal SYBR Green (Bio-Rad) in a 15 μL final volume containing 6 μL diluted template cDNA and specific primers (0.5 μM) (Supplementary Table S1). Amplification was performed on a CFX96 Touch real-time PCR system (Bio-Rad) with the following conditions: 95°C for 7 min and 40 cycles at 95°C for 10 s and 60°C for 40 s. Amplification was followed by a melt curve analysis. Absolute quantification of transcript copy number was assessed with calibration curves. Transcript levels were then normalized with EF1α.

### Subcellular Localization Experiments

Subcellular localization of MdCHK receptors were studied in *Catharanthus roseus* C20D cells transiently transformed using plasmid-coated particles bombardment as described in Guirimand et al. (2009). The full length MdCHK sequences were amplified and cloned into the *SpeI* restriction site of pSCA-YFP plasmid (for primers, see Supplementary Table S1), in frame with the 5′ extremity of the YFP coding sequence. The endoplasmic reticulum (ER) cyan fluorescent protein (CFP) marker (Guirimand et al., 2010) was used in co-transformation assays.

Dynamic localization of MdCHK receptors was also studied in yeast *S. cerevisiae* strain WT303 (*MAT*a/α, *leu2, trp1, ura3, ade2, his3*) transformed by pESC-LEU plasmids (Foureau et al., 2016) containing *MdCHK* sequences, except for MdCHK2, cloned in pYES2 and transformed in *sln1*Δ yeast strain to bypass the sequence toxicity in microorganisms. The CYP450 T16H2 sequence was clone in the pESC-TRP plasmid in fusion with the 5′end of the CFP sequence and used as an ER marker (Besseau et al., 2013). *MdCHK* sequences were cloned under galactose inducible promoter and fused at the 5′ end with the YFP sequence. Transformed colonies were cultivated on selective plates (CSM-LEU or CSM-URA, supplemented by 2% glucose, respectively, for pESC-LEU and pYES2) at 30°C for 48 h and then transferred in inducing liquid media (CSM-LEU or CSM-URA, supplemented by 2% galactose, respectively, for pESC-LEU and pYES2) with or without iP (5 μM) for additional overnight culture at 28°C.

An Olympus BX51 epifluorescence microscope equipped with the Olympus DP71 digital camera and Cell^∗^D imaging software (Soft Imaging System Olympus) was used for image capture and merging false-colored images of both C20D cells and *S. cerevisiae* colonies expressing YFP.

### BiFC Interaction Assays

Bimolecular Fluorescent Complementation (BiFC) experiments were conducted using SPYNE and SPYCE plasmids (Waadt and Kudla, 2008). MdCHK sequences were amplified and cloned into the *SpeI* restriction site (for primers, see Supplementary Table S1), in frame with the 5′ extremity of a truncated YFP coding sequence. Transient transformation of *C. roseus* cells by particle bombardment and YFP imaging were performed according to Guirimand et al. (2009) with adaptation for BiFC assays (Guirimand et al., 2010). Interactions were tested in triplicates using three independent plasmid clones.

### RNA-seq Data Analysis

Available RNAseq data for *M. domestica* was downloaded from NCBI via SRA toolkit2.6.2. The recovered SRA files were transformed in fastq format with the “fastq-dump” command from SRA toolkit. The files were cleaned with Trimmomatic 0.36 with default parameters and using provided adapter sequences for TruSeq2 and TruSeq3. The transcription quantification was performed with Salmon 0.6.1 using the Variational Bayesian EM algorithm and biase correction. TPM (transcripts per million) values from the resulting quant.sf files were combined under R 3.3.0 in an expression matrix containing 95,232 predicted genes (*Malus domestica* v3.0) × 250 experimental conditions. Using the expression matrix, Pearson Correlation Coefficients (PCC) and further Highest Reciprocal Ranks (HRR) computation were performed using a homemade program written in C [HRR (gene A, gene B)] = max [rank (gene A, gene B), rank (gene B, gene A)] to establish the co-expressed genes lists for MdCHK and GO enrichment tests. For each *MdCHK* highly co-expressed genes, i.e., genes with a HRR ≤ 500 were selected. The procedure was repeated with publicly available *A. thaliana* RNAseq data. We similarly prepared an expression matrix containing 33,604 transcripts (*Arabidopsis* TAIR v10 genome annotation) and 1,676 samples. Co-expressed genes lists were obtained after calculating PCC and ranking them with HRR. For each AtCHK (AT5G35750.1, AT1G27320.1, AT2G01830.2), gene pairs having an HRR < 500 were considered to be significantly co-expressed. Orthology between *Arabidopsis* and apple tree was obtained from Plaza 3.0 (Van Bel et al., 2012). The functions represented by coexpressed genes of each MdCHK were analyzed with the Gene Ontology classification. A BlastX was performed on the *M. domestica* genome v3.0 to recover correspondent protein sequences and Pfam domains were identified using Hmmer. The functional annotations of the *M. domestica* genome v3.0 were generated using Trinotate on the previous data. In order to determine potential functional enrichment for every target gene, enrichment of GO terms was tested by comparing effectives to a hypergeometric distribution (*p*-value cut-off = 0.001) using the R “phyper” function. To compare redundancies in the five co-expressed genes lists, a Venn diagram was drawn using the venneuler package 1.1 for R.

## Results

### Identification of Five *Malus domestica* CHASE-Containing Histidine Kinases (MdCHKs)

**FIGURE 1:**
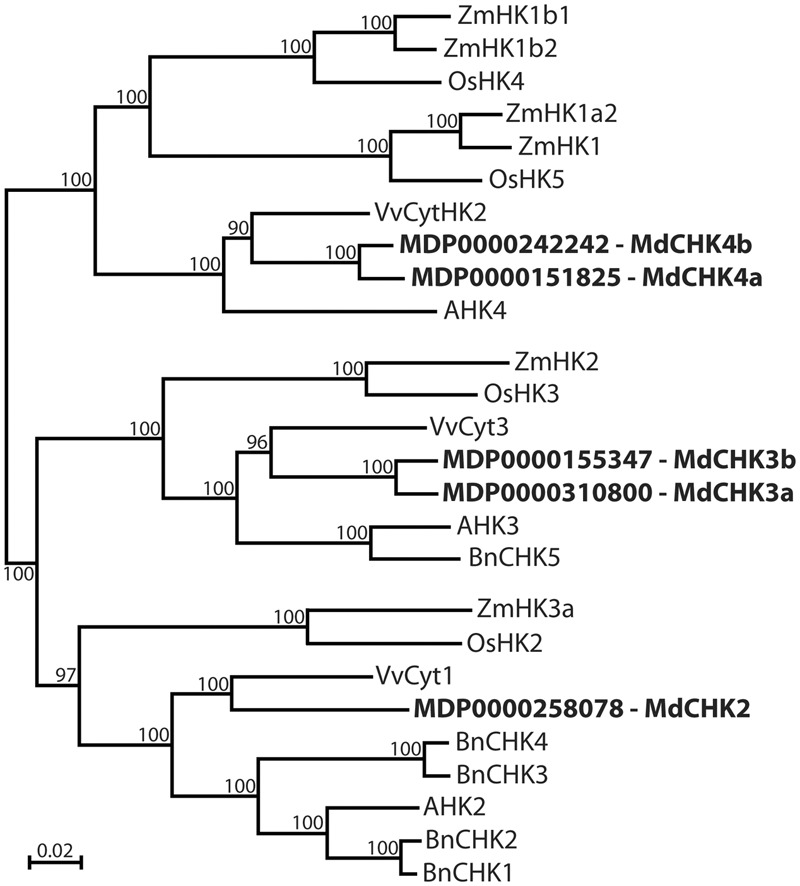
Phylogenetic analysis of *Malus domestica* cytokinin receptors. Apple tree CHASE Histidine Kinase receptors MdCHK2 (KM114879), MdCHK3a (KM114880), MdCHK3b (KM114881), MdCHK4a (KM114883), and MdCHK4b (KM114882) were compared to cytokinin receptors characterized in angiosperms. The tree was constructed by neighbor-joining distance analysis on conserved protein sequence domains. *Brassica napus*: BnCHK1 (KF621029), BnCHK2 (KF621030), BnCHK3 (KF621031), BnCHK4 (KF621032), BnCHK5 (KF621033); *Arabidopsis thaliana*: AHK2 (At5g35750), AHK3 (At1g27320), AHK4 (At2g01830); *Oryza sativa*: OsHK5 (Os02g50480), OsHK2 (Os10g21810), OsHK3 (Os01g69920), OsHK4 (Os03g50860); *Vitis vinifera*: *VvCyt3* (*CAO42401*), *VvCyt1* (GSVIVT01030058001), *VvCyt2* (*CAO66151*); *Zea mays*: ZmHK1 (NP_001104859*)*, ZmHK2 (NP_001104866), ZMHK3a (AB102957), ZmHK1a2 (NP_001105857), ZmHK1b1 (NP_001105858), ZmHK1b2 (NP_001105913).

Based on the CHASE domain of the three *A. thaliana* CHKs, apple tree genome was browsed to identify putative *CHK* sequences in *M. domestica*. Five candidates were identified and named *MdCHK2* (locus tag *MDP0000258078*), *MdCHK3a* (locus tag *MDP0000310800*), *MdCHK3b* (locus tag *MDP0000155347*), *MdCHK4a* (locus tag *MDP0000151825*) and *MdCHK4b* (locus tag *MDP0000242242*) according to their distribution within the three classical groups homologous to*AHK2, AHK3*, and *AHK4*, as shown by phylogenetic analysis (**Figure 1**). Genomic sequences revealed that *MdCHK2* possesses 13 exons and is located on chromosome 9 (Supplementary Figure S1). Both *MdCHK3a* and *MdCHK3b*, respectively, located on chromosomes 16 and 13, display 10 exons with a similar organization regarding intron positions and intron/exon sizes. The same observation was made for *MdCHK4a* and *MdCHK4b* respectively located on chromosomes 13 and 10 and containing 11 exons. Such similarity may reflect the gene duplication events leading to the couples of CHKs homologous *MdCHK3a*/*MdCHK3b* and *MdCHK4a*/*MdCHK4b* which share respectively 94.67 and 95.67% nucleotide identity. The full-length cDNAs of the five MdCHKs were cloned and deposited at NCBI under the GenBank accession numbers KM114879 to KM114883. They contained large open-reading frames ranging from 3027 to 3612 bp encoding proteins of 1008 to 1203 amino acids.

**FIGURE 2:**
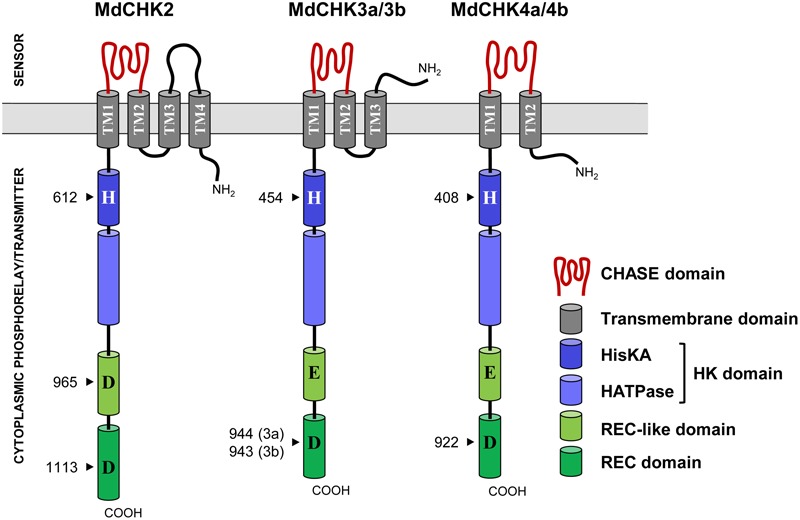
Domain structure of the five MdCHK receptors. MdCHK receptors display a classical CHK organization with a CHASE sensing domain, transmembrane domains (TM), a histidine kinase (HK) domain composed of a HK dimerization and phosphoacceptor domain (HisKA) and a HK catalytic domain (HATPase), a receiver (REC)-like domain and a REC domain. The conserved residues of each domain are indicated (arrows).

The computational analysis of the protein sequences of MdCHKs revealed the presence of the four basic conserved domains called Cyclases/Histidine kinases Associated SEnsory (CHASE), Histidine Kinase (HK), Receiver (REC), and Receiver-like (REC-like) (**Figure 2** and Supplementary Figure S2). While these modular MdCHKs share a similar multidomain architecture in their cytoplasmic C-terminal part (HK, REC-like and REC domains), they differ in the TM domain topology of the N-terminus part. Indeed, the MdCHK4a and MdCHK4b have two predicted α-helices transmembrane domains TM1 and TM2 (Supplementary Figure S3) bordering the CHASE domain (**Figure 2**). MdCHK3a and MdCHK3b possess equivalent TM1/CHASE/TM2 organization with an additional predicted transmembrane helice (TM3) in N-terminus. Finally, the N-terminus of MdCHK2 includes a fourth predicted transmembrane domain (TM4). Consequently, in addition of the extracytoplasmic loop containing the sensing CHASE domain, the TM3 and TM4 domains border a supplemental extracytoplasmic loop (140 aa) absent in MdCHK3a/b and MdCHK4a/b structures (**Figure 2**). Regarding the cytoplasmic part, each MdCHK possesses an HK domain containing the conserved phosphorylatable histidine residue as well as the C-terminal REC domain including the conserved phospho-accepting aspartate residue (**Figure 2** and Supplementary Figure S2). In addition, the five receptors contain a second receiver domain located between the HK domain and the REC domain called pseudo-receiver domain or REC-like (**Figure 2**). Interestingly, the putative phospho-accepting aspartate residue in the REC-like domain of MdCHK2 is conserved, suggesting that this domain may be functional in terms of phosphorelay reaction, whereas the corresponding residues in MdCHK3a/MdCHK3b and MdCHK4a/MdCHK4b are substituted with glutamate (Supplementary Figure S2).

### MdCHKs Function as Cytokinin Receptors in a Yeast Complementation Assay

**FIGURE 3:**
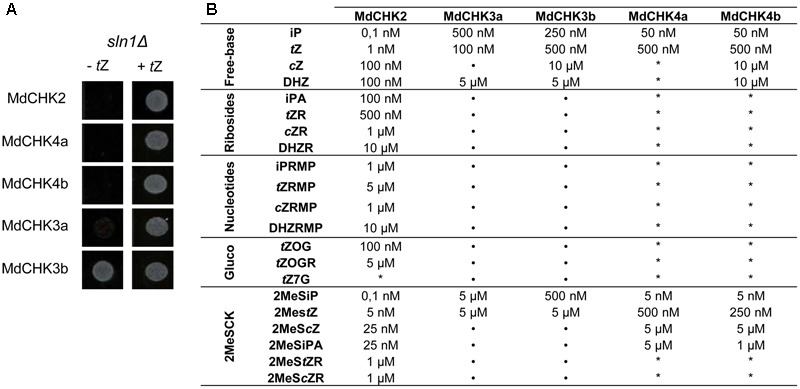
*sln1*Δ mutated yeast complementation assays with MdCHK. **(A)** Cytokinin-dependant growth phenotype of mutant yeast *sln1*Δ transformed with pYES2-MdCHK. Suspensions of transformed yeasts were spotted onto plates containing or not *t*Z (10 μ M). **(B)** Cytokinin binding specificity and sensitivity profiles of MdCHKs. MdCHK-complemented yeasts were grown in liquid culture medium containing different cytokinin types with different concentrations for 48 h. Minimum concentration inducing yeast growth is indicated for each cytokinin tested. iP, isopentenyladenine; *t*Z, *trans*-zeatin; cZ, *cis*-zeatin; DHZ, dihydrozeatin; iPA, isopentenyladenosine; *t*ZR, *trans*-zeatin riboside; *c*ZR, *cis*-zeatin riboside; DHZR, dihydrozeatin riboside; iPRMP, isopentenyladenosine 5′-monophosphate; *t*ZRMP, *trans*-zeatin riboside 5′-monophosphate; cZRMP, *cis*-zeatin riboside 5′-monophosphate; DHZ, dihydrozeatin riboside 5′-monophosphate; *t*ZOG, *trans*-zeatin *O*-glucoside; *t*ZOGR; *trans*-zeatin *O*-glucoside riboside; *t*Z7G, *trans*-zeatin N7-glucoside; 2MeSiP, 2-methylthio-isopentyladenine; 2MeS*t*Z, 2-methylthio-*trans*-zeatin; 2MeS*c*Z, 2-methylthio-*cis*-zeatin; 2MeSiPA, 2-methylthio-isopentyladenosine; 2MeS*t*ZR, 2-methylthio-*trans*-zeatin riboside; 2MeS*c*ZR, 2-methylthio-*cis*-zeatin riboside. ^∗^, no growth observed. Basal growth of MdCHK3a- and MdCHK3b-complemented yeast is also indicated (•).

To assess the function of the five MdCHKs as cytokinin receptors, we exploited the *S. cerevisiae sln1*Δ deletion mutant strain which carries a lethal mutation in the *SLN1* gene encoding its unique osmosensing histidine kinase (Inoue et al., 2001). The *sln1*Δ yeast mutants carrying pYES2-MdCHK2, pYES2-MdCHK4a or pYES2-MdCHK4b were lethal (**Figure 3A**). However, the addition of *trans*-zeatin (*t*Z) in culture medium allowed recovering a normal growth (**Figure 3A**). By depending on the presence and perception of cytokinin to complement *sln1* mutation, the three recombinant yeast strains clearly demonstrated that MdCHK2, MdCHK4a, and MdCHK4b act as cytokinin receptors in this heterologous system. Concerning the recombinant strains carrying pYES2-MdCHK3a and pYES2-MdCHK3b, they displayed an original phenotype since they exhibited a basal growth in absence of cytokinin, especially for MdCHK3b (**Figure 3A** and Supplementary Figure S4). However, the addition of *t*Z clearly induced the yeast growth pointing out that both MdCHK3a and MdCHK3b sense cytokinins (**Figure 3A** and Supplementary Figure S4). The basal constitutive activity of these two cytokinin receptors raised the question of their putative additional sensing function.

### MdCHK Receptors Show Different Binding Specificities toward Cytokinin

We further evaluated the substrate specificity and sensitivity of *M. domestica* cytokinin receptors. In this way, we measured the growth of the yeast cells in presence of various cytokinin-types at different concentrations including free-bases, ribosides, glucosides, and methylthio cytokinins. The specificity as well as the sensitivity (the minimal cytokinin concentration that induced yeast growth) were reported on **Figure 3B** and Supplementary Figure S4. Three distinct profiles corresponding to MdCHK2, MdCHK3, and MdCHK4 groups were observed. First, MdCHK2 clearly perceived a wide range of cytokinin forms since each cytokinin type activated the receptor except *t*Z7G. Furthermore, this receptor presented a remarkable higher sensitivity than other MdCHKs (until 0,1 nM for iP and 2MeSiP forms) and was the only one to be activated by some ribosides and glucosides cytokinin-types. Secondly, the strictly cytokinin-dependent MdCHK4a and MdCHK4b were activated by the free-bases iP and *t*Z and the methylthio-forms 2MeSiP, 2MeS*t*Z, 2MeS*c*Z, and 2MeSiPA (Supplementary Figure S4). High concentrations (10 μM) of *c*Z and DHZ were also effective on MdCHK4b. Nevertheless, the cytokinin-sensitivity of both MdCHK4a and MdCHK4b was obviously lower than MdCHK2. Finally, even if MdCHK3a and MdCHK3b showed a basal growth in absence of cytokinin, we were able to detect a significant difference of growth in presence of iP, *t*Z, DHZ, 2MeSiP, and 2MeS*t*Z (Supplementary Figure S4).

### *MdCHKs* Exhibit Distinct Expression Patterns

**FIGURE 4:**
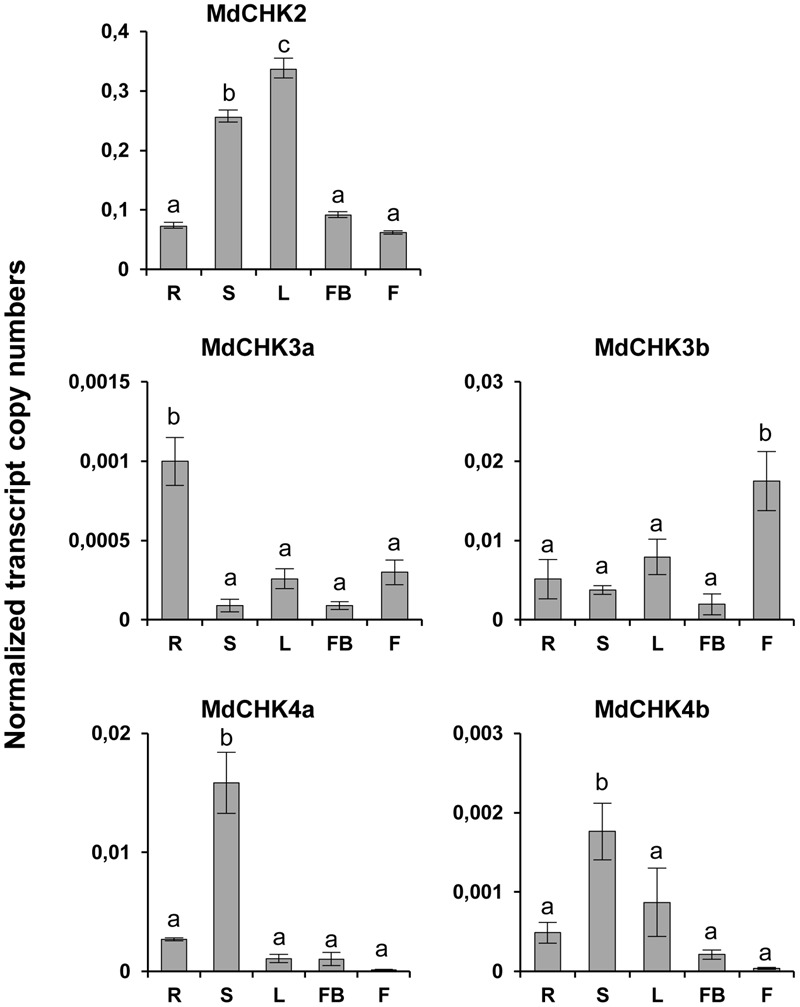
*MdCHKs* display various expression patterns in apple tree organs. Total transcript numbers of *MdCHK2, MdCHK3a, MdCHK3b, MdCHK4a*, and *MdCHK4b* were determined by real-time RT-PCR analyses performed on total RNA extracted from various *M. domestica* organ*s*. R, roots; S, stems; L, leaves; FB, flower buds; F, flowers. Transcript abundance of each gene was normalized against *EF1*α reference gene. Data have been analyzed by ANOVA (*p* < 0.05), after running Shapiro–Wilk and Bartlett tests, followed by HSD Tukey test. Error bars were calculated from triplicates.

To examine the gene expression of *MdCHKs*, RT-qPCR was carried out using distinct plant organs including roots, stems, leaves, flower buds and flowers. Transcripts of the five *MdCHKs* were detected in all the tested organs, but with distinct expression pattern. Thus, *MdCHK2* reached higher expression level in leaves and stems (**Figure 4**). *MdCHK4a* and *MdCHK4b* displayed similar expression profiles with high expression level in stems whereas gene expression was hardly detected in flowers (**Figure 4**). *MdCHK3a* and *MdCHK3b* disclosed differential pattern of expression. While *MdCHK3a* was mainly expressed in roots, *MdCHK3b* showed its highest expression in flowers (**Figure 4**). Finally, substantial differences in the overall expression level of each *MdCHKs* were also observed. Interestingly, *MdCHK2* displayed the higher expression level whilst *MdCHK3b/MdCHK4a* and *MdCHK3a/MdCHK4b* retained a 10- and a 100-fold lower expression, respectively.

**FIGURE 5:**
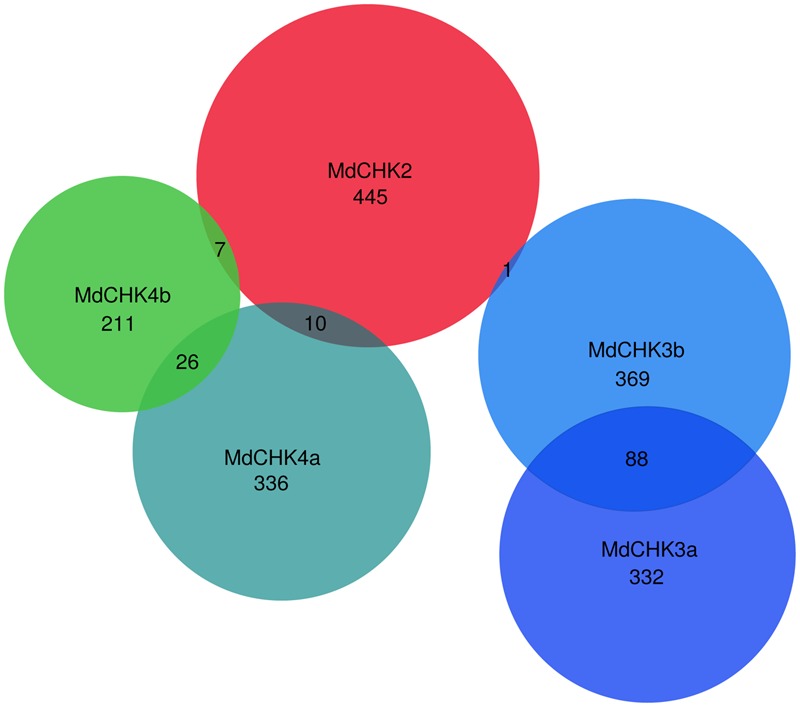
Venn diagram of MdCHK co-expressed gene lists. Best co-expressed gene lists (HRR < 500) were separately established for each MdCHK and compared (see Materials and Methods).

**FIGURE 6:**
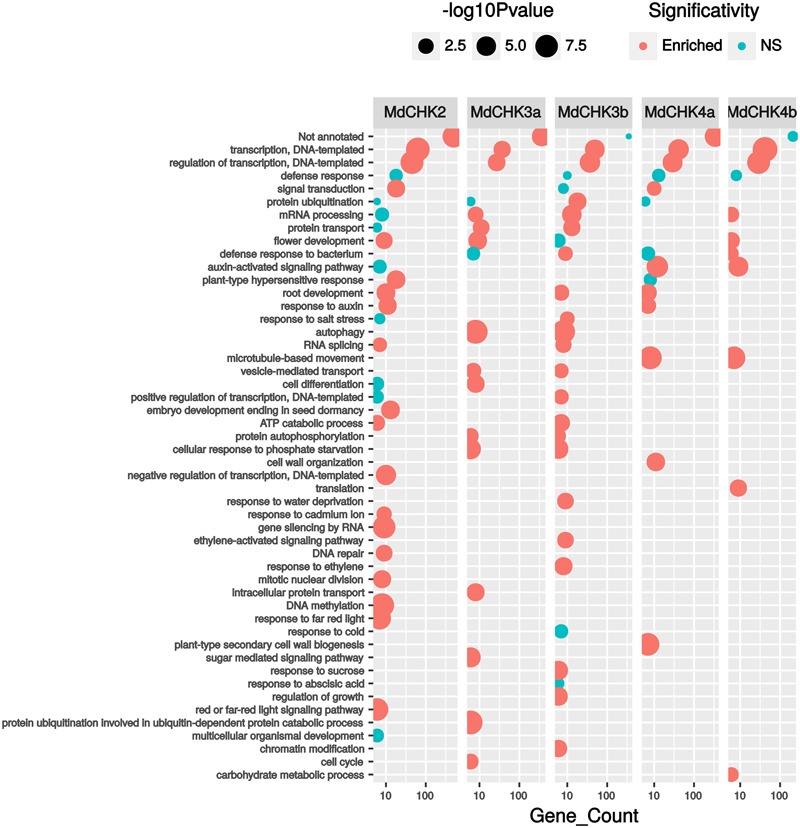
Functional annotation of best co-expressed genes (HRR < 500) with Gene Ontology (GO) terms. Only GO terms from ≪ Biological process ≫ represented by 5 or more genes were kept. *P*-values correspond to GO term enrichment tests which were performed by comparison to hypergeometrical distribution.

To complete qPCR analysis, we used the available RNAseq data to generate an expression matrix of apple tree genes (Supplementary Table S2). The best co-expressed genes with each *MdCHK* through our expression matrix were investigated and compared (Supplementary Table S3). A very weak degree of overlap among lists of genes co-expressed with each *MdCHK* was found. *MdCHK2* shared up to 18 genes with other cytokinin receptors. *MdCHK3* and *MdCHK4* groups did not have common co-expressed genes. *MdCHK3a/MdCHK3b* homologs as well as *MdCHK4a/MdCHK4b* homologs shared respectively 88 and 26 genes (**Figure 5**). This low overlapping of co-associated genes might support the limited functional redundancy of MdCHKs. Each MdCHK co-expressed genes list was compared to the list established for their *Arabidopsis* ortholog in order to highlight potential shared genes. We found a relatively weak overlap between functions associated to either CHK (Supplementary Figure S5A and Table S4). For example, only 19 genes were similarly co-expressed between *MdCHK2* (1.8%) and *AtCHK2* (8.7%). While such a weak overlap could be due to the initial datasets used to calculate correlations which differ in size and experiments, conserved co-expressed genes may be good candidates for a further investigation of the cytokinin pathway. In addition, we found very small overlaps between co-expressed gene lists of *AtCHKs*, as observed for *MdCHKs*, reinforcing a potential specificity in CHK functions (Supplementary Figure S5B). Enrichment tests of Gene Ontology (GO) terms performed on each list of co-expressed genes also gave an overview of possible specific physiological processes associated with each receptor. For example, “Embryo development ending in seed dormancy” and “Response to cadmium ion” were exclusively enriched for *MdCHK2* whereas “Plant-type secondary cell wall biogenesis” and “Regulation of growth” were specifically enriched for *MdCHK4a* and *MdCHK3b*, respectively (**Figure 6** and Supplementary Figure S6).

### MdCHKs Mainly Localize to the Endoplasmic Reticulum in Plant and Show a Dynamic Behavior in Response to Cytokinin in Yeast

**FIGURE 7:**
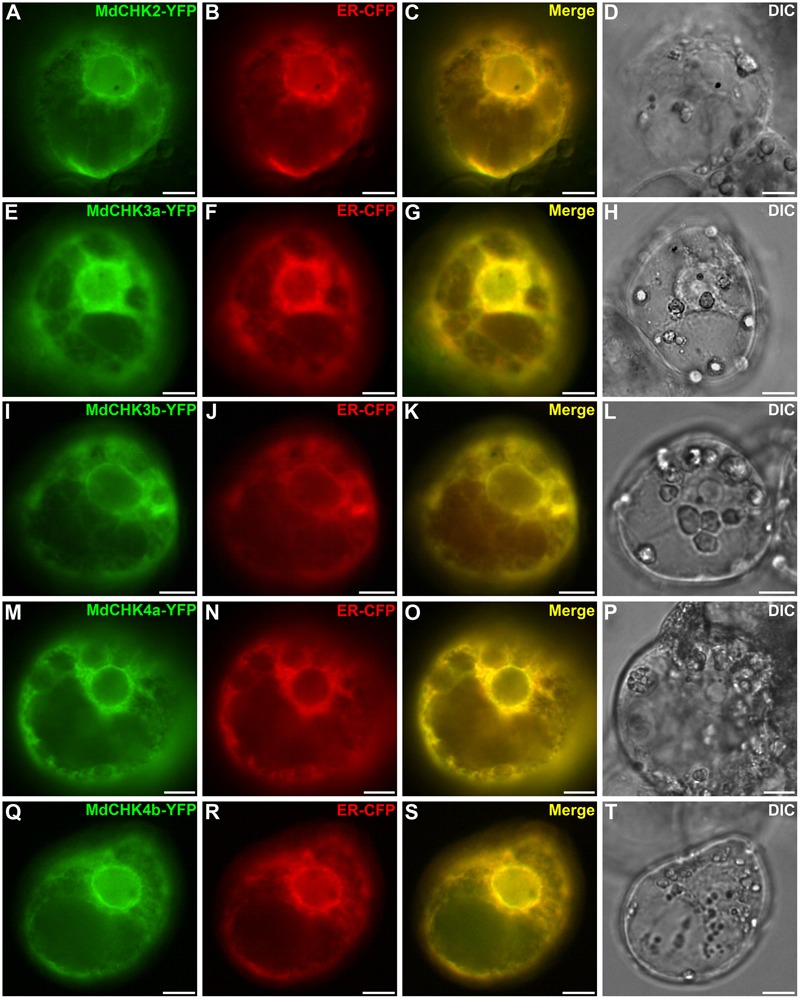
MdCHK-YFP fusion proteins are localized at the Endoplasmic Reticulum (ER) membrane. *Catharanthus roseus* cells were transiently co-transformed with plasmids expressing MdCHK-YFP **(A,E,I,M,Q)** and endoplasmic reticulum-CFP marker **(B,F,J,N,R)**. Co-localization of the two fluorescence signals appeared on the merged image **(C,G,K,O,S)**. The morphology is observed with differential interference contrast (DIC; **D,H,L,P,T**). Scale bars: 10 μm.

We investigated the subcellular distribution of the MdCHKs using C-terminal YFP tagging to ensure the correct anchoring of the transmembrane domains. MdCHK-YFP constructs were transiently expressed in *C. roseus* cells that constitute a reliable model for studying protein subcellular localization (Foureau et al., 2016). In transiently transformed cells, the fusion proteins displayed a fluorescence signal located in the endoplasmic reticulum (ER) network throughout the cell as well as in the perinuclear space (**Figures 7A,E,I,M,Q**). The signal perfectly co-localized (**Figures 7C,G,K,O,S**) with the specific ER-CFP marker (**Figures 7B,F,J,N,R**), confirming that the five MdCHKs are located in the ER of plant cells. Noteworthy, cytokinin addition in plant cell medium did not alter the ER localization of the MdCHK-YFP fusions (data not shown).

**FIGURE 8:**
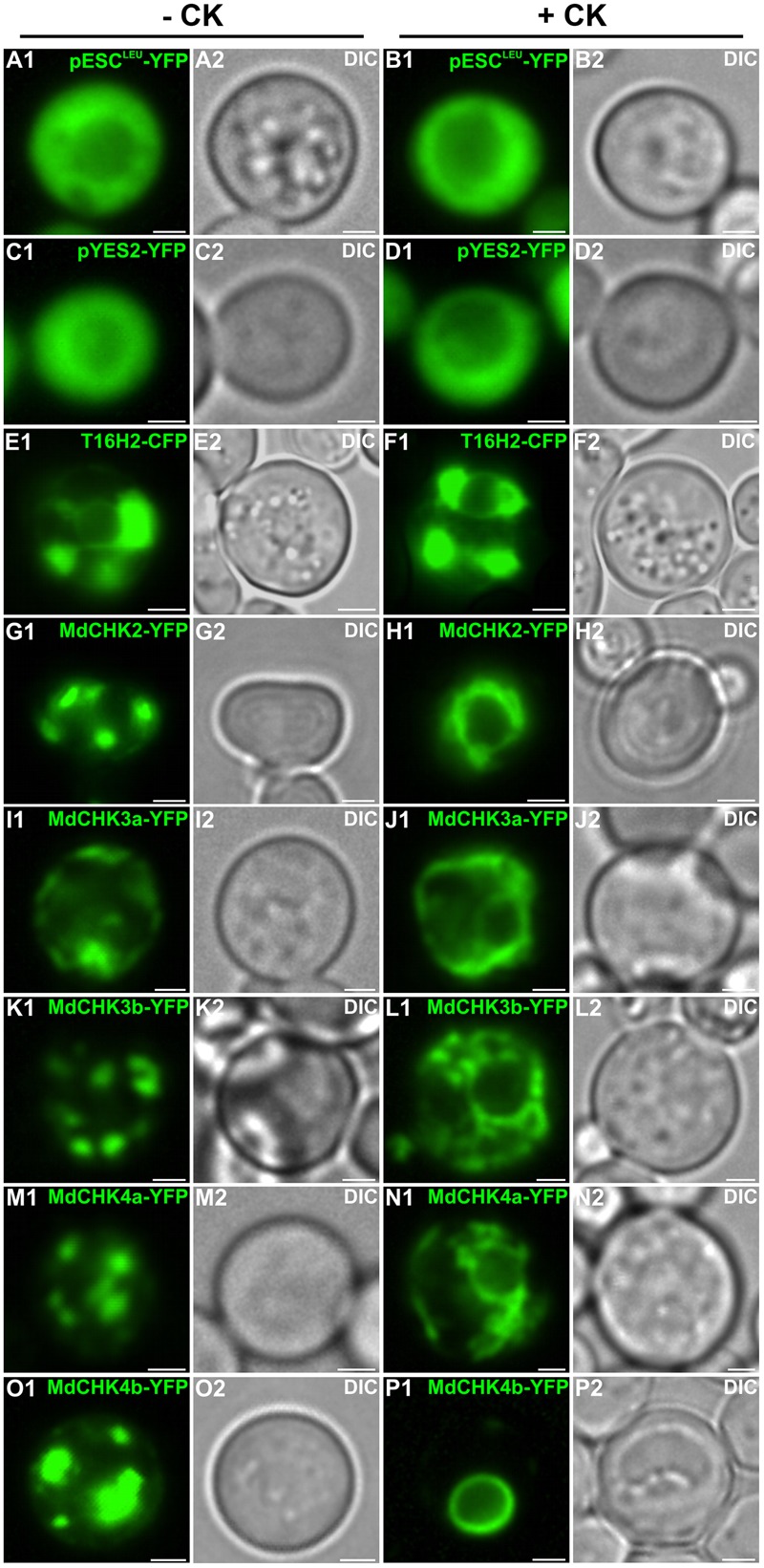
Dynamic localization of the MdCHK receptors at the Endoplasmic Reticulum membrane of *S. cerevisiae* in response to cytokinins. *Saccharomyces cerevisiae* cells were transformed with plasmids expressing MdCHK-YFP fusion proteins and grown in liquid culture media containing or not iP (5 μM). Fluorescence signals displayed by the fusion proteins are shown **(G1–P1)**. As controls, *S. cerevisiae* cells were transformed with the empty vectors pESC^Leu^-YFP and pYES2-YFP **(A1–D1)**. The T16H2-CFP serves as an ER marker **(E1,F1)**. The morphology is observed with differential interference contrast (DIC; **A2–P2**). Scale bars: 2 μm.

Since plant cells may produce their own pool of cytokinins preventing the study influence of exogenous cytokinins on the localization of the MdCHKs, we therefore investigated the subcellular distribution of MdCHKs in the yeast *S. cerevisiae*, by using YFP fusion proteins. In absence of cytokinins, a punctate fluorescence pattern was observed for the five MdCHKs (**Figures 8G1,I1,K1,M1,O1**), which accumulated in the ER forming structures comparable as organized smooth ER (Snapp et al., 2003) that is described to result from protein interactions. Upon cytokinin treatment, the ER localization of the five MdCHKs did not change. But interestingly, a reorganization of the fluorescent pattern was observed reflecting the decrease or disappearance of aggregate structures (**Figures 8H1,J1,L1,N1,P1**). Indeed, MdCHK2 and MdCHK4b displayed a strong perinuclear localization (**Figures 8H1,P1**). Concerning MdCHK3a, MdCHK3b, and MdCHK4a, the fluorescence signal appeared in a discontinuous pattern as well as in the perinuclear space (**Figures 8J1,L1,N1**). In order to ensure that cytokinins themselves had no impact on the architecture of the ER, we used T16H2-CFP construct as a specific ER marker (Besseau et al., 2013). Upon cytokinin treatment, no redistribution of fluorescence signal was observed, reinforcing the plausibility of a specific reorganization of MdCHKs in response to cytokinin signal (**Figures 8E1,F1**).

### Homo- and Heterodimerization Are Common Features of MdCHK2 and MdCHK4 in Contrast to the MdCHK3 Pair That Only Displays Specific Heterodimerization Characteristics

**FIGURE 9:**
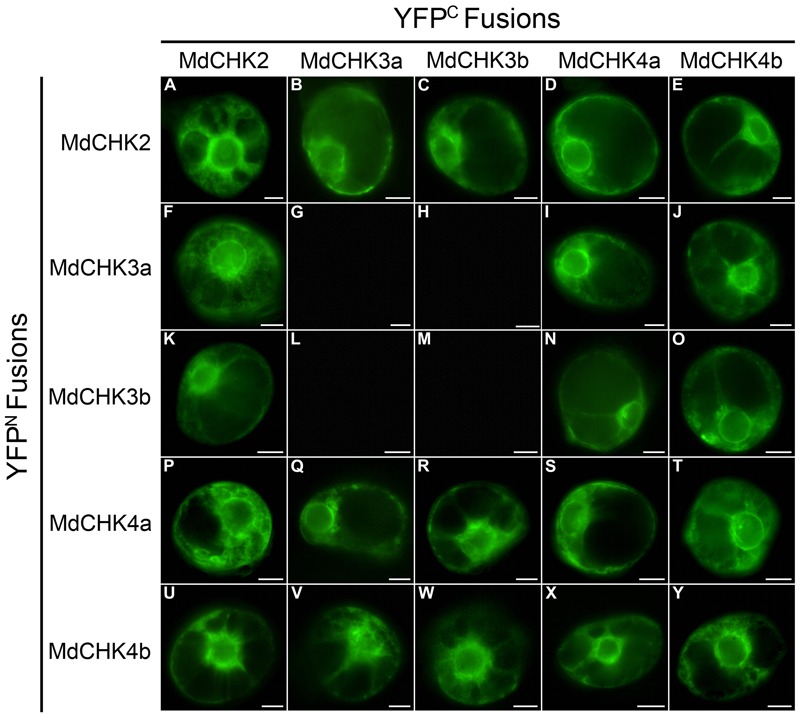
MdCHKs interact to form homodimers and heterodimers in BiFC assays. Cells of *C. roseus* were transiently co-transformed using the plasmids expressing the different MdCHK proteins fused with the YFP^N^ and YFP^C^ split in C-terminal. Homodimerizations **(A,G,M,S,Y)** and heterodimerizations **(B–F,H–L,N–R,T–X)** have been tested, with both YFP^N^ and YFP^C^ combinations. Three independent plasmid clones were used to test interactions. Scale bars: 10 μm.

Cytokinin receptors were previously proposed to interact each other to enable the *trans*-phosphorylation of the HK domain after cytokinin perception (Dortay et al., 2006; Caesar et al., 2011; Hothorn et al., 2011; Wulfetange et al., 2011). Considering the multiple possibilities of interactions between the five MdCHKs, homo- and hetero-dimerization were investigated by BiFC assays *in planta*. The full coding sequences of MdCHKs were cloned upstream of the coding sequence of the two split-YFP fragments (YFP^N^ and YFP^C^) to generate the MdCHK-YFP^N^ and MdCHK-YFP^C^ fusion proteins. BiFC analysis revealed that MdCHK2, MdCHK4a, and MdCHK4b were able to form homodimers within the ER network (**Figures 9A,S,Y**) whereas no BiFC complex reconstitution was observed when testing the MdCHK3a and MdCHK3b homodimers (**Figures 9G,M**). Moreover, no signal was detected with the MdCHK3a and MdCHK3b heterodimer combination (**Figures 9H,L**). Additionally, a cytokinin application did not result in the formation of a fluorescent signal within the three configurations (data not shown). Nevertheless, MdCHK3a and MdCHK3b were able to heterodimerize with MdCHK2, MdCHK4a and MdCHK4b within the ER (**Figures 9B,C,F,I–K,N,O,Q,R,V,W**). In addition, MdCHK2 and both MdCHK4 homologs shape heterodimers with each other (**Figures 9D,E,P,U,T,X**).

## Discussion

Five CHASE domain-containing Histidine Kinases belonging to the three classical groups were identified in *M. domestica*: *MdCHK2*, the homolog of *Arabidopsis AHK2* and the two pairs *MdCHK3a*/*MdCHK3b* and *MdCHK4a*/*MdCHK4b*, homologous to *AHK3* and *AHK4*, respectively. These two pairs might be the direct consequence of apple genome-wide duplication (Velasco et al., 2010) as it was found in another hardwood tree *Populus trichocarpa* (Nieminen et al., 2008).

To confirm the functionality of MdCHKs as cytokinin receptors, we conducted a cytokinin-responsive assay based on the use of the *sln1*Δ *S. cerevisiae* strain mutant (Maeda et al., 1994). We thus showed that MdCHK2, MdCHK4a, and MdCHK4b restore the viability of the *sln1*Δ mutant in a strictly cytokinin-dependent fashion providing convincing evidences of their cytokinin receptor function. By contrast, both MdCHK3a and MdCHK3b, they presented an unexpected profile since they conferred a basal growth to the *sln1*Δ mutant in absence of cytokinin. However, the enhancement of the yeast growth in presence of cytokinin confirmed their cytokinin receptor function. It is important to emphasize that the basal constitutive activity confers an originality for MdCHK3a and MdCHK3b compared to MdCHK2, MdCHK4a, and MdCHK4b. To our knowledge, a constitutive activity for a CHK protein has never been reported before.

The extensive exploitation of our five MdCHK-complemented yeast strains revealed that the receptors differed greatly in their cytokinin specificity and sensitivity. Three distinct specificity and sensitivity profiles clearly emerged. The most remarkable result comes from MdCHK2 which perceives an unprecedented range of cytokinins including nucleotide-type precursors as well as free-base-, riboside-, *O*-glucoside- and methylthio-forms, with a substantial sensitivity for iP, *t*Z, 2MeSiP, and 2MeS*t*Z (**Figure 3B** and Supplementary Figure S4). Until now, due to its presumed toxicity in bacteria, few studies examined the ligand-binding properties of the full-length *Arabidopsis* AHK2 and its homologs in other plant species. Recently, an *E. coli* cytokinin-binding assay revealed that the full-length BnCHK1 and BnCHK3, two AHK2 homologs in *Brassica napus*, showed high affinity for *t*Z, iP, and *t*ZR (Kuderová et al., 2015). A tobacco membrane assay also revealed that *t*Z and iP strongly interacted with AHK2, whereas their conjugated forms did not, suggesting that free bases were the sole biologically active cytokinin compounds (Lomin et al., 2015). Even if our experiments used a heterologous system in which the yeast membrane environment can potentially differ from those of plant, we highlighted not only the receptor ability to bind hormones but also their activation through cytokinin perception activating the phosphorelay in yeast. Thus, we can assume that our assay reflects the biological activity of cytokinin riboside and nucleotide forms on the receptor. Moreover, artefactual cytokinin activation or conversion occurrence in yeast can be omitted, since MdCHK4a and MdCHK4b are not activated in presence of the nucleotide-, riboside- or *O*-glucoside types. Therefore, the broad cytokinin spectrum of MdCHK2 raises the question of its possible central role in *M. domestica*. Such hypothesis is also reinforced by the high expression level of MdCHK2 compared to other MdCHKs (**Figure 4**). Concerning MdCHK4a and MdCHK4b, they perceived a restricted spectrum of cytokinins such as free-base forms and some of the methylthiolated-forms with a lower sensitivity than MdCHK2 supporting previous works obtained with *Arabidopsis* AHK4 and AHK2 (Stolz et al., 2011). Concerning MdCHK3a and MdCHK3b, they perceived some cytokinin free-bases and methylthiolated forms. However, regarding their constitutive activity, we must consider that we could have under estimated their real cytokinin binding effectiveness and that it might not really reflect their complete capacity to perceive the diverse structures of cytokinins. The original activities of MdCHK3a and MdCHK3b were definitely interesting and need to be further investigated. In particular, did the monomeric or heterodimeric forms of MdCHK3a/b influence their cytokinin perception? In any case, our results clearly supported previous works reporting that cytokinin-binding properties of AHK3 differed from those of AHK2 and AHK4 (Spíchal et al., 2004; Romanov et al., 2006; Stolz et al., 2011; Heyl et al., 2012).

These different properties between MdCHK receptors raised the question of their specialized functions in apple tree. MdCHKs clearly showed an organ-specific gene expression pattern (**Figure 4**) and the analysis of gene co-expression profiles with each MdCHK unequivocally shed light on the weak degree of overlap among lists of co-expressed genes that strongly underlines the distinct roles of the five receptors in physiological processes (**Figure 5** and Supplementary Figure S6). Besides these distinct patterns, we can point out that both pairs of homologs MdCHK3 and MdCHK4 might acquire distinct functions after duplication.

From a structural point of view, the five MdCHKs possess the conserved CHASE, HK, REC-like and REC domains also found in *Arabidopsis*, maize and rapeseed (Ueguchi et al., 2001; Yonekura-Sakakibara et al., 2004; Kuderová et al., 2015). While the architecture of cytoplasmic C-terminal part is similar within MdCHK, the topology of their N-terminus differs in the number of predicted transmembrane domains which surround the CHASE sensing domain (**Figure 2**). MdCHK4a/MdCHK4b and MdCHK3a/MdCHK3b possess two and three transmembrane domains, respectively, whereas MdCHK2 exhibits a fourth transmembrane helix that forms a unique additional extracytoplasmic loop. This variability might reflect the specific sensing activities of MdCHK receptors. It is well established that structural variations in the CHASE domain result in different ligand specificities of *Arabidopsis* receptors (Romanov et al., 2006; Heyl et al., 2007; Stolz et al., 2011). Nevertheless, the organization of the surrounding environment of the CHASE domain might also be important for receptor functioning (Steklov et al., 2013). Indeed, the CHASE flanking regions including transmembrane helices are assumed to play a substantial role in localization and intramolecular signaling (Steklov et al., 2013). Directed mutagenesis on a transmembrane helix highlighted its importance for the AHK4 receptor activation (Miwa et al., 2007). Moreover, experiments on the CHASE domain of AHK4 compared with the full-length receptor revealed significant differences in the binding affinity, highlighting the importance of the CHASE environment in cytokinin perception (Stolz et al., 2011). Thus, it cannot be excluded that architectural variations in the N-terminal part of the MdCHKs somehow influence their distinct properties.

Regarding the cytoplasmic C-terminal part, only MdCHK2 harbors a phospho-accepting aspartate in the receiver-like domain (Supplementary Figure S2). Its presence is a common feature shared with AHK2 and the four AHK2 homologs in *B. napus* BnCHK1, BnCHK2, BnCHK3, and BnCHK4 (Ueguchi et al., 2001; Kuderová et al., 2015). However, the function of this receiver-like domain has not been yet elucidated. As reported above, MdCHK2 perceives a broad cytokinin spectrum with a substantial sensitivity compared to other MdCHKs, thus it would be interesting to further investigate if the second phosphorylatable aspartate contribute to the properties of this receptor. Indeed, if we consider that the MdCHK2 receiver-like domain is functional, it might optimize the phosphorelay reaction in addition to the receiver domain or might guide specific interactions with downstream HPts.

As previously described in other species, the five MdCHKs are located at the ER membrane and the perinuclear space. Moreover, we revealed for the first time, a dynamic redistribution of cytokinin receptors in response to cytokinin application. The development of an approach in *S. cerevisiae* allowed us to overcome the use of plant cells, which probably produce their own cytokinins and prevent studying cytokinin influence. More precisely, we showed that MdCHKs relocalized through the ER network, especially by getting closer to the nucleus (**Figure 8**). This result clearly supports the current concept of phosphotransfer enhancement through a perinuclear localization which overcomes intracellular distance and optimizes the signal transduction (Caesar et al., 2011; Wulfetange et al., 2011).

We also report herein a complete analysis of the full-length cytokinin receptors interactions *in planta*. Until now, only homodimerization of full-length AHK2 was demonstrated *in planta* (Wulfetange et al., 2011) and a partial study in yeast two-hybrid system based on full-length receptors showed the AHK3/AHK4 interaction as well as the formation of AHK3 homo-oligomers (Caesar et al., 2011). Here, we examined the homo- and the hetero-dimerization of the MdCHKs *in planta* since histidine kinases are supposed to act as dimers. Not only MdCHK2, MdCHK4a, and MdCHK4b homodimerize, but they form heterodimers with each other. Surprisingly, MdCHK3a and MdCHK3b do not form homodimers. This feature might explain their singularity in cytokinin perception in our yeast system compared to MdCHK2, MdCHK4a, and MdCHK4b. Furthermore, MdCHK3a and MdCHK3b did not heterodimerize with each other, but exclusively heterodimerize with MdCHK2, MdCHK4a, and MdCHK4b. This particularity needs to be deeply addressed for the complete understanding of MdCHK3 functioning. To date, our overview clearly emphasized the complexity of cytokinin perception in *M. domestica* since MdCHKs are able to form not only homodimers, but also heterodimers as well as monomers. Regarding the heterodimers in plant cells, they provided a new layer of intricacy since most of the histidine kinases form homodimers in order to autophosphorylate (Capra and Laub, 2012). Nevertheless, their physiological relevance *in planta* needs to be further determined. It cannot be excluded that CHK heterodimers might operate in cytokinin perception contributing to specify the cytokinin signaling pathways in order to regulate distinct physiological processes. For instance, the low gene expression of the MdCHK3 and MdCHK4 pairs compared to MdCHK2 suggested a MdCHK2 dimerization ratio in favor of homodimerization. Such homodimers could ensure signaling for the major physiological processes associated to cytokinins while MdCHK2 heterodimers would be associated to more discrete functions. In this way, the putative functions of MdCHK3 predicted through gene correlation analysis (**Figure 6**) would be assumed by heterodimers with MdCHK2 or MdCHK4 since the MdCHK3 pair does not homo- or heterodimerize and is potentially not able to active the phosphorelay.

## Conclusion

This work provided a framework for further functional studies of cytokinin receptors in apple tree. In particular, it will be greatly interesting to focus on their involvement in response to the pathogens of apple tree. Furthermore, a structural approach would also contribute to gain insights into the key aspects of the mechanisms by which MdCHKs are differentially activated by cytokinin signal.

## Author Contributions

DD, EA, CM, and GG conducted experiments. FL and TDdB achieved bioinformatics analyses. NP, VC, AO, AL, MCl, OP, and SB participated in the design of the study and interpretation. DG, SC, NG-G, MCo, JC, and SB assisted in the supervision of this work. GG conceived, supervised and coordinated the work. DD and GG wrote the first draft of the manuscript, to which all authors contributed.

## Conflict of Interest Statement

The authors declare that the research was conducted in the absence of any commercial or financial relationships that could be construed as a potential conflict of interest.

## Footnotes

1 http://www-nmr.cabm.rutgers.edu/bioinformatics/Proteomic_tools/Helical_wheel/
