# Supplementary material for: CHASE-Containing Histidine Kinase Receptors in Apple Tree: From a Common Receptor Structure to Divergent Cytokinin Binding Properties and Specific Functions
Source: Front Plant Sci. 2017 Sep 20;8:1614. doi: 10.3389/fpls.2017.01614 (PMC5611679; doi:10.3389/fpls.2017.01614)
Supplement: Supplementary file 1 [file Presentation_1.PDF]

frontiers

*Supplementary Material*

# CHASE-containing histidine kinase receptors in apple tree: from a common structure to divergent cytokinin binding properties and specific functions

Dimitri Daudu, Elsa Allion, Franziska Liesecke, Nicolas Papon, Vincent Courdavault, Thomas Dugé de Bernonville, Céline Mélin, Audrey Oudin, Marc Clastre, Arnaud Lanoue, Martine Courtois, Olivier Pichon, David Giron, Sabine Carpin, Nathalie Giglioli-Guivarc'h, Joël Crèche, Sébastien Besseau, Gaëlle Glévarec.

Corresponding author: Gaëlle Glévarec
gaelle.glevarec@univ-tours.fr

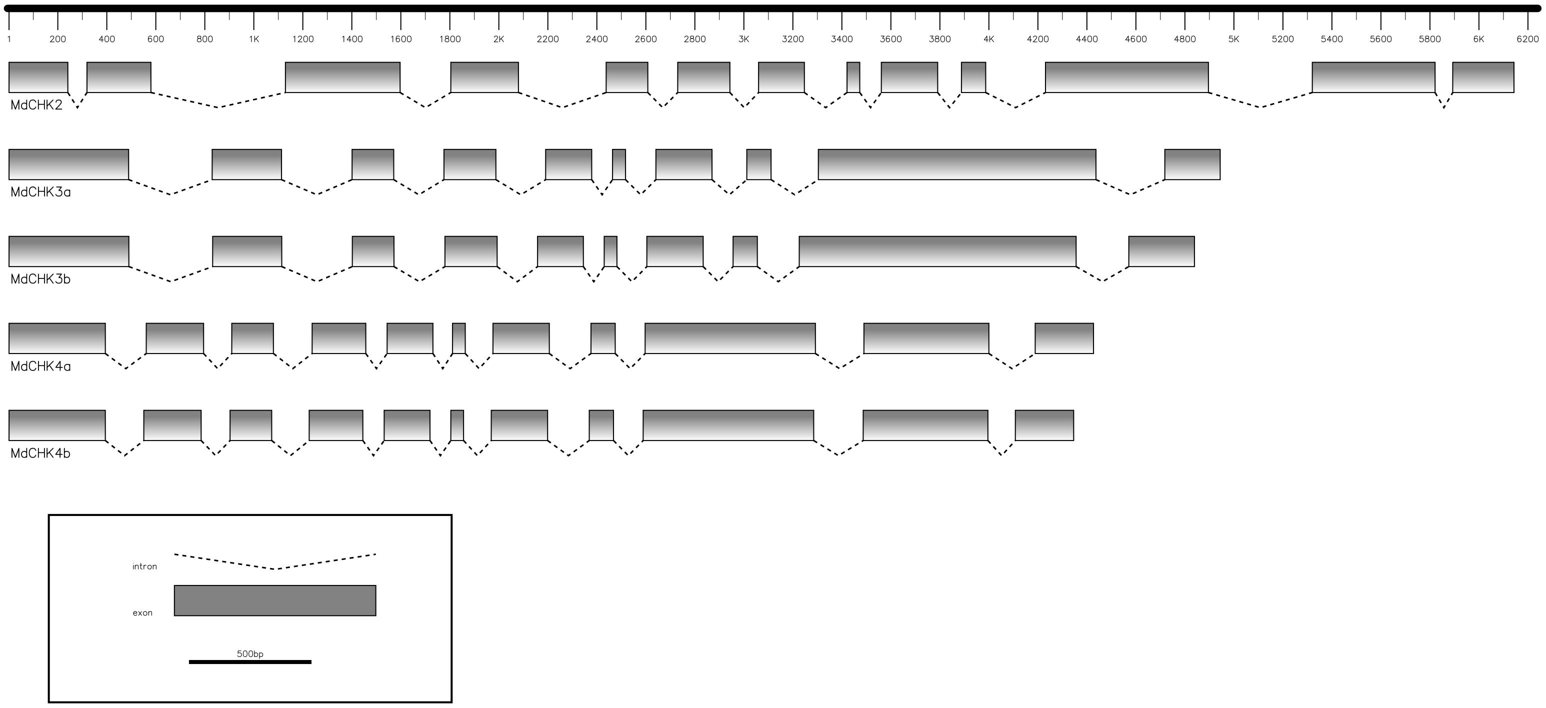


**Supplementary Figure S1.** Gene structure of MdCHK. Exons and introns are indicated by boxes and solid lines, respectively (http://bio.ieo.eu/fancygene).

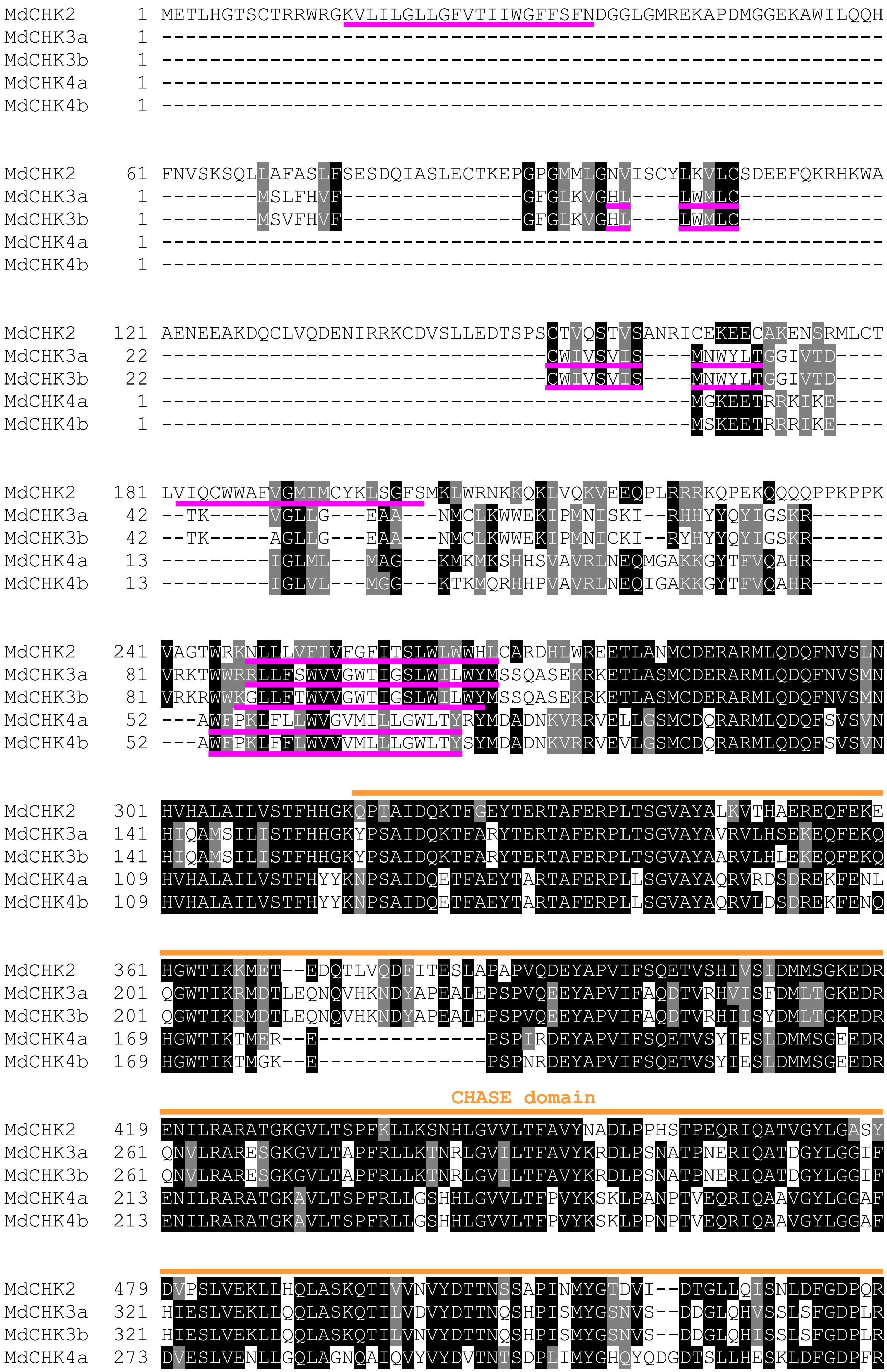
MdCHK2 1 METLHGTSCTRRWRGKVLILGLLGFVTIIWGFFSFNDGGLGMREKAPDMGGEKAWILQQH
MdCHK3a 1 ------------------------------------------------------------
MdCHK3b 1 ------------------------------------------------------------
MdCHK4a 1 ------------------------------------------------------------
MdCHK4b 1 ------------------------------------------------------------
MdCHK2 61 FNVSKSQLLAFASLFSESDQIASLECTKEPGPGMMLGNVISCYLKVLCSDEEFQKRHKWA
MdCHK3a 1 ---------MSLFHVF--------------GFGLKVGHL----LWMLC------------
MdCHK3b 1 ---------MSVFHVF--------------GFGLKVGHL----LWMLC------------
MdCHK4a 1 ------------------------------------------------------------
MdCHK4b 1 ------------------------------------------------------------
MdCHK2 121 AENEEAKDQCLVQDENIRRKCDVSLLEDTSPSCTVQSTVSANRICEKEECAKENSRMLCT
MdCHK3a 22 --------------------------------CWIVSVIS----MNWYLTGGIVTD----
MdCHK3b 22 --------------------------------CWIVSVIS----MNWYLTGGIVTD----
MdCHK4a 1 --------------------------------------------MGKEETRRKIKE----
MdCHK4b 1 --------------------------------------------MSKEETRRRIKE----
MdCHK2 181 LVIQCWWAFVGMIMCYKLSGFSMKLWRNKKQKLVQKVEEQPLRRRKQPEKQQQQPPKPPK
MdCHK3a 42 --TK-----VGLLG---EAA---NMCLKWWEKIPMNISKI--RHHYYQYIGSKR------
MdCHK3b 42 --TK-----AGLLG---EAA---NMCLKWWEKIPMNICKI--RYHYYQYIGSKR------
MdCHK4a 13 ---------IGLML---MAG---KMKMKSHHSVAVRLNEQMGAKKGYTFVQAHR------
MdCHK4b 13 ---------IGLVL---MGG---KTKMQRHHPVAVRLNEQIGAKKGYTFVQAHR------
MdCHK2 241 VAGTWRKNLLLVFIVFGFITSLWLWWHLCARDHLWREETLANMCDERARMLQDQFNVSLN
MdCHK3a 81 VRKTWWRRLLFSWVVGWTIGSLWILWYMSSQASEKRKETLASMCDERARMLQDQFNVSMN
MdCHK3b 81 VRKRWWKGLLFTWVVGWTIGSLWILWYMSSQASEKRKETLASMCDERARMLQDQFNVSMN
MdCHK4a 52 ---AWFPKLFLLWVGVMILLGWLTYRYMDADNKVRRVELLGSMCDQRARMLQDQFSVSVN
MdCHK4b 52 ---AWFPKLFFLWVVVMLLLGWLTYSYMDADNKVRRVEVLGSMCDQRARMLQDQFSVSVN
MdCHK2 301 HVHALAILVSTFHHGKQPTAIDQKTFGEYTERTAFERPLTSGVAYALKVTHAEREQFEKE
MdCHK3a 141 HIQAMSILISTFHHGKYPSAIDQKTFARYTERTAFERPLTSGVAYAVRVLHSEKEQFEKQ
MdCHK3b 141 HIQAMSILISTFHHGKYPSAIDQKTFARYTERTAFERPLTSGVAYAARVLHLEKEQFEKQ
MdCHK4a 109 HVHALAILVSTFHYYKNPSAIDQETFAEYTARTAFERPLLSGVAYAQRVRDSDREKFENL
MdCHK4b 109 HVHALAILVSTFHYYKNPSAIDQETFAEYTARTAFERPLLSGVAYAQRVLDSDREKFENQ
MdCHK2 361 HGWTIKKMET--EDQTLVQDFITESLAPAPVQDEYAPVIFSQETVSHIVSIDMMSGKEDR
MdCHK3a 201 QGWTIKRMDTLEQNQVHKNDYAPEALEPSPVQEEYAPVIFAQDTVRHVISFDMLTGKEDR
MdCHK3b 201 QGWTIKRMDTLEQNQVHKNDYAPEALEPSPVQEEYAPVIFAQDTVRHIISYDMLTGKEDR
MdCHK4a 169 HGWTIKTMER--E--------------PSPIRDEYAPVIFSQETVSYIESLDMMSGEEDR
MdCHK4b 169 HGWTIKTMGK--E--------------PSPNRDEYAPVIFSQETVSYIESLDMMSGEEDR
CHASE domain
MdCHK2 419 ENILRARATGKGVLTSPFKLLKSNHLGVVLTFAVYNADLPPHSTPEQRIQATVGYLGASY
MdCHK3a 261 QNVLRARESGKGVLTAPFRLLKTNRLGVILTFAVYKRDLPSNATPNERIQATDGYLGGIF
MdCHK3b 261 QNVLRARESGKGVLTAPFRLLKTNRLGVILTFAVYKRDLPSNATPNERIQATDGYLGGIF
MdCHK4a 213 ENILRARATGKAVLTSPFRLLGSHHLGVVLTFPVYKSKLPANPTVEQRIQAAVGYLGGAF
MdCHK4b 213 ENILRARATGKAVLTSPFRLLGSHHLGVVLTFPVYKSKLPPNPTVEQRIQAAVGYLGGAF
MdCHK2 479 DVPSLVEKLLHQLASKQTIVVNVYDTTNSSAPINMYGTDVI--DTGLLQISNLDFGDPQR
MdCHK3a 321 HIESLVEKLLQQLASKQTILVDVYDTTNQSHPISMYGSNVS--DDGLQHVSSLSFGDPLR
MdCHK3b 321 HIESLVEKLLQQLASKQTILVNVYDTTNQSHPISMYGSNVS--DDGLQHISSLSFGDPLR
MdCHK4a 273 DVESLVENLLGQLAGNQAIQVYVYDVTNTSDPLIMYGHQYQDGDTSLLHESKLDFGDPFR

```
MdCHK4b    273  DIESLVENLLGQLAGNQAIQVYVYDVTNTSDPLIMYGHQYQDGDTSLLHESKLDFGDPYR

MdCHK2     537  KHEMHCRFKHKPPFPLTAASASIGVLVITFLVSHIFHAAISRIAKVEADYREMMELKVRA
MdCHK3a    379  NHEMRCRFKQKPPWPWLAITTSIGILVIALLVGYIFHATVNRIAKVEDDFHKMMELKKQA
MdCHK3b    379  NHEMRCRFKHKPPWPWLAITTSFGILVIAFLVGYIFHATVNRIAKVEDDFRKMMELKKQA
MdCHK4a    333  RHQMICRYHQTAPMSWTAVNTAFLFFVIGFLVGYILYGAAMHIVKVEDDFREMEELKVRA
MdCHK4b    333  KHQMICRYHQRAPTSWTAINTAFLFCVIGFLVGYILYGAAMHIVKVEDDFREMEELKVRA

MdCHK2     597  EAADVAKSQFLATVSHEIRTPMNGVLGMLQMLMDTDLGPNQQDYAETAHASGRELISLIN
MdCHK3a    439  EAADVAKSQFLATVSHEIRTPMNGVLGMLHMLMDTNLDVTQLDYVRTAQGSGKALVSLIN
MdCHK3b    439  EAADVAKSQFLATVSHEIRTPMNGVLGMLHMLMDTNLDVTQLDYVRTAQGSGKALVSLIN
MdCHK4a    393  EAADVAKSQFLATVSHEIRTPMNGILGMLALLLDTALSSTQMDYARTAQACGKALITLIN
MdCHK4b    393  EAADVAKSQFLATVSHEIRTPMNGILGMLALLLDTALSSTQMDYARTAQACGKALITLIN

MdCHK2     657  EVLDQAKIESGRLELETVPFDLRSVLDNVLSLNSGKSNEKGIELAAYVSNMVPEVVIGDP
MdCHK3a    499  EVLDQAKIESGKLELEAVRFDLRAILDDVLSLFSGKSQQKGVELAVYISDQVPDMLIGDP
MdCHK3b    499  EVLDQAKIDSGKLELEALRFDLRAILDDVLSLFSGKSQEKGVELAVYISDQVPDMLIGDP
MdCHK4a    453  EVLDRAKIDAGKLELEEVPFGIRSILDDVLSLFSEKTRNKGIELAVFVSDKVPEIFMGDP
MdCHK4b    453  EVLDRAKIDAGKLELEEVPFGIRSILDDVLSLFSEKSRNKGIELAVFVSDKVPDIFMGDP
```

HK domain

```
MdCHK2     717  GRFRQIITNLVGNSIKFTHDKGHIFVSVHLVDEVRAPPDLMDEVLRQGFNLVGDVSN--K
MdCHK3a    559  GRFRQIITNLMGNSIKFT-EKGHIFVTVHLVEELIGSIGVETES---------------S
MdCHK3b    559  GRFRQIITNLIGNSIKFT-EKGHIFVTVHLVEELIGSIDVETES---------------S
MdCHK4a    513  GRFRQIITNLVGNSIKFT-ERGHIFVKVHLAEPSKVMINGKSETYLNGGPDEGVLTSDGH
MdCHK4b    513  GRFRQIITNLVGNSIKFT-ERGHIFVKVHLAEPSKVMINGKSETYLNGGSDEGVLTSDGH

MdCHK2     775  TYNTLSGFPVVNRWKSWEGFNSI------RSTTL-EKSDMIKLLVTVEDTGVGIPLDAQN
MdCHK3a    603  SKNTLSGFPVADKHRSWGGFRCFGQDG--SASRFSSSSDLINIIVSVEDTGVGIPLEAQS
MdCHK3b    603  SKNTLSGFPVADRRRSWGGFRCFGQDG--STNQF-SSSDLINIIVSVEDTGVGIPLEAQS
MdCHK4a    572  QFKTLSGCEAANDWNSWDTFKHLVSDEEYRADVD-EASEKVTLMVSVEDTGIGIPLGAQE
MdCHK4b    572  QFKTLSGCEAANDWNSWDKFKHLVSDEEYRADVV-EASEQVTLMVSVEDTGIGIPLAAQE

MdCHK2     828  RIFTPFMQADSSTSRTYGGTGIGLSISKRLVDLMSGEIGFVSEPGTGSTFSFMGTFGKGE
MdCHK3a    661  RVFTPFMQVGPSISRTHGGTGIGLSISKCLVGLMKGEIGFVSIPKIGSTFTFTAVFTNAS
MdCHK3b    660  RVFTPFMQVGPSISRTHGGTGIGLSISKCLVGLMKGEIGFVSIPKIGSTFTFTAVFTNVS
MdCHK4a    631  RVFMPFMQADSSTSRHYGGTGIGLSISKCLVELMGGQIKFVSRPHIGSTFSFTANFRRCK
MdCHK4b    631  RVFMPFMQADSSTSRHYGGTGIGLSISKCLVELMGGQIKFVSLPRVGSTFSFTANFRRCK

MdCHK2     888  LSSL-ETK----WHQYEPAVSEFQGLRALVIDNKTIRTEVTKYHMQRLGISVDTASCLES
MdCHK3a    721  SSSN-ELTIEQINSQSNAASSEFNGMTALVVDQRPVRAKMSSYHIERLGIRVEVVSDLNQ
MdCHK3b    720  SNSN-ELTIQQMNSQSNAASSEFNGMTALVVDQRPVRAKMSRYHIQRLGICVEVVSDLNQ
MdCHK4a    691  KNAFSDMK----KPNPEDLPSSLRGLRAIVVDRKLVRAAVTKYHLKRLGIVVEVASSIKM
MdCHK4b    691  KNAFSDMK----KPNPEDLPSSFRGLRAIVVDGKLVRAAVTKYHLKRLGILVEVASSIKM
```

REC-like domain

```
MdCHK2     943  ACSYLSSAGNTSLSTPLA--MVLIDKDVWDKETGIILHQSLKEHRKNNVELLINLPKILL
MdCHK3a    780  GLASISCGSTTIN-------MVLVEQEVWNKDSGTSALFVSNLRKIDG-----QVPPKLF
MdCHK3b    779  GLSSIISGNTTIN-------MVLVEQEVWDKDSSTSALFVNNLRKIDG-----QVPLKLF
MdCHK4a    747  AVASCGRNGSVASGNSIQPDIVLVEKDSWISGEEHDLNAQKLDWKQNGHAF--KLPKMIL
MdCHK4b    747  AVASCGRNGAAASGKSIQPDIVLVEKDSWISGEEHDLNVQKLDWKQNGHAF--KLPKMIL

MdCHK2    1001  VAT-SITSVERNVVTSLGLVDKVLTKPLRLSVLIACFQEALG----RRKKRLLNANKPTL
```

```
MdCHK3a   828 ILANSSSSCRISSATSGVSTPTVIMKPLRASMLAASLQRAMGVG---NKGNLRNGELPSL
MdCHK3b   827 ILTNSISSCRISSATSGVSTPTVIMKPLRASMLAASLQRAMGVG---NKGNLRNGDLPSL
MdCHK4a   805 LATKNLSQAESDNVRAAGFADTVIMKPLRASMVAACLQEVLGIGKKRQPGRVVPNGSNVL
MdCHK4b   805 LATNNLSQAESDKVRAAGFADTVIMKPLRASMVAACLQEVLGIGKKRQPGRVVPNGSNVL

MdCHK2   1056 --GKLLREKRILVVDDNVVNRRVAEGALKKYGAIVTCVESGKVALLKLKPPHNFDACFMD
MdCHK3a   885 SLRNLLLGRKILIIDDNNVNLRVAAGALKKYGAEVICADSGKKAISLLTPPHHFDACFMD
MdCHK3b   884 SLRNLLLGRKILIIDDNNVNLRVAAGALKKYGAEVVCADSGKKAISLLTPPHHFDACFMD
MdCHK4a   865 --QSLLCGKKILVVDDNLVNRRVAAGALKKFGANVECVDSGKAALALLQLPHNFDACFMD
MdCHK4b   865 --QSLLCGKKILVVDDNLVNRRVAAGALKKFGANVECVDSGKAALALLQLPHNFDACFMD

                                   REC domain
MdCHK2   1114 LQMPEMDGFEATRIIRGMESEVNEKIAAGEASVEMFGNVVNWHTPILAMTADVIQASNEE
MdCHK3a   945 IQMPEMDGFEATRRIRDLERNISN-------------SIQAWHVPILAMTADVIQATHEE
MdCHK3b   944 IQMPEMDGFEATRRIRNMECNISNHIQHGEVSAEDYENIQAWHVPILAMTADVIQATHEE
MdCHK4a   923 IQMPEMDGFEATRRIRQMESKANVEMNGGFEGLTTKGD---WHLPVLAMTADVIHATYDE
MdCHK4b   923 IQMPEMDGFEATRRIRQMESKANVETNGGFEALARRGD---WHLPVLAMTADVIHATYDE

MdCHK2   1174 CIKCGMDDYVSKPFEEDQLYSAVARFFESG------
MdCHK3a   992 CTKCGMDGYVSKPFEAEQLYREVSRFFQSTTTGNA-
MdCHK3b  1004 CTRCGMDGYVSKPFEAEQLYREVSRFFQSTTTGSLE
MdCHK4a   980 CLKCGMDGYVSKPFEEENLYQAVAKFFKT-------
MdCHK4b   980 CLKCGMDGYVSKPFEEENLYQAVAKFFKT-------
```

**Supplementary Figure S2.** Alignment of MdCHK amino-acid sequences. Membrane-spanning sequences are underline in pink, CHASE in orange, Histidine-Kinase in green, Receiver-like domain in blue and Receiver domain in red. Conserved histidine residue of the HK domain and aspartate residue of REC and REC-like domains are highlighted in green, red and blue, respectively.

# MdCHK2

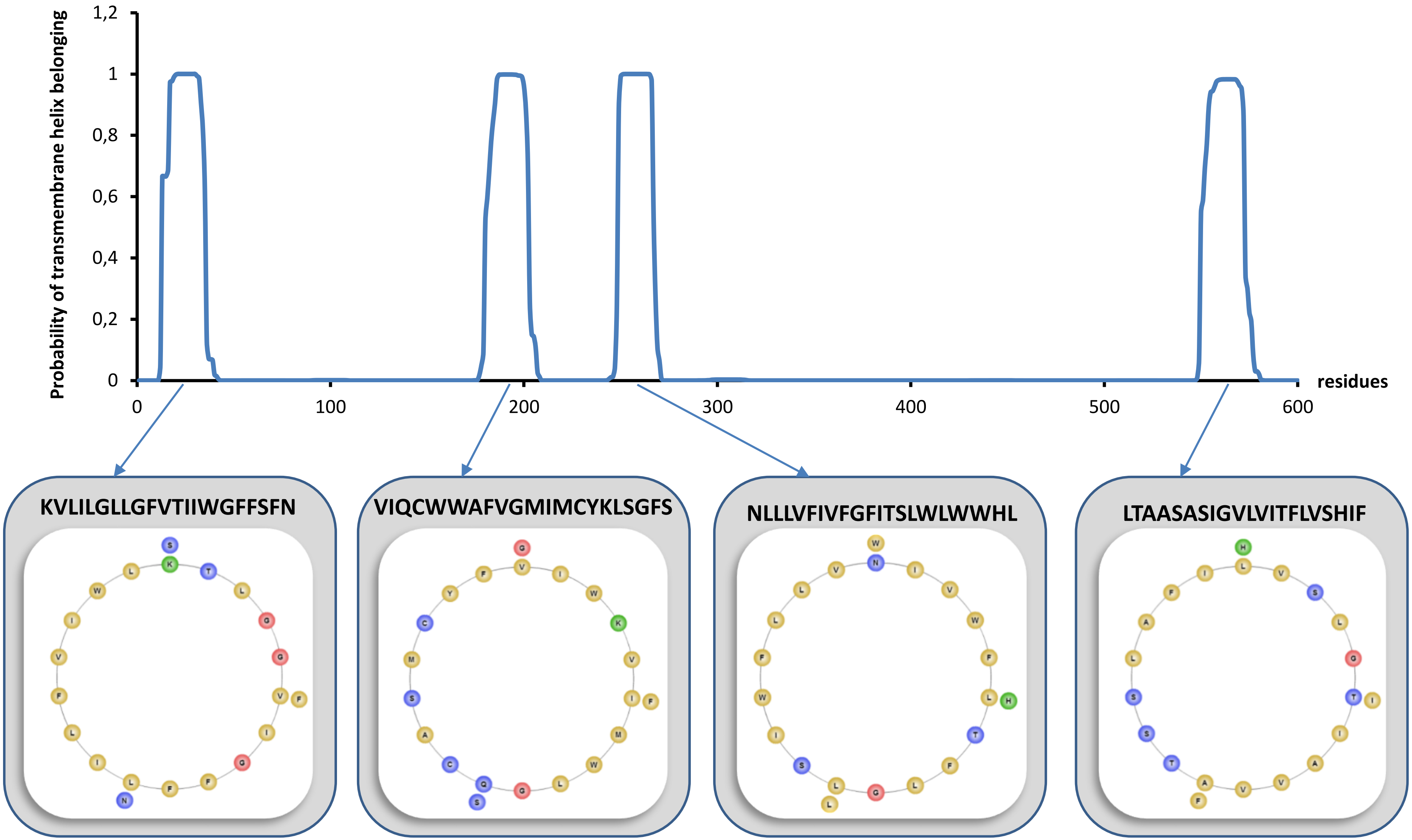

Probability of transmembrane helix belonging
1,2
1
0,8
0,6
0,4
0,2
0
0
100
200
300
400
500
600
residues
KVLILGLLGFVTIIWGFFSFN
VIQCWWAFVGMIMCYKLSGFS
NLLLVFIVFGFITSLWLWWHL
LTAASASIGVLVITFLVSHIF

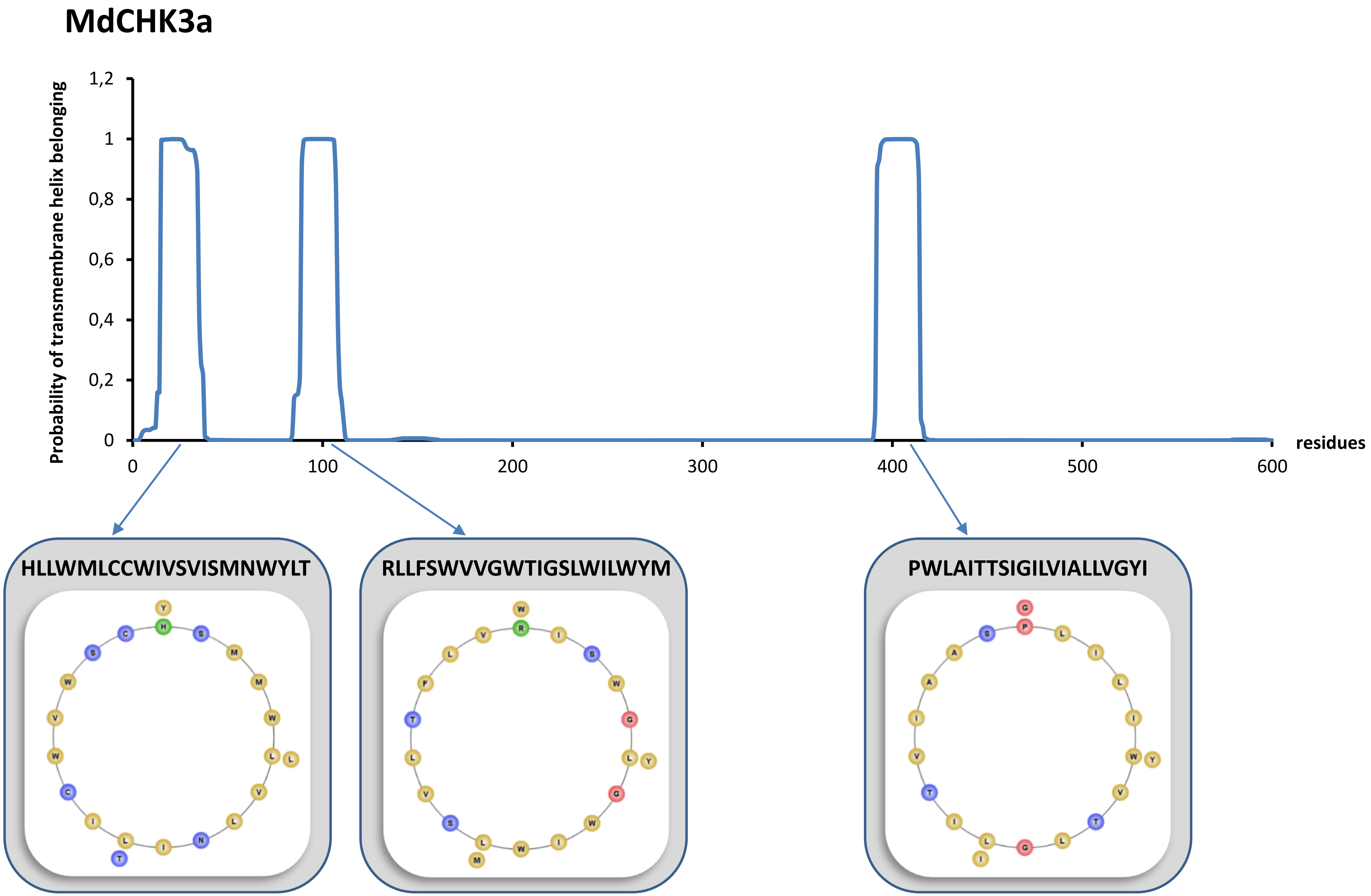
MdCHK3a
Probability of transmembrane helix belonging
1,2
1
0,8
0,6
0,4
0,2
0
0
100
200
300
400
500
600
residues
HLLWMLCCWIVSVISMNWYLT
RLLFSWVVGWTIGSLWILWYM
PWLAITTSIGILVIALLVGYI

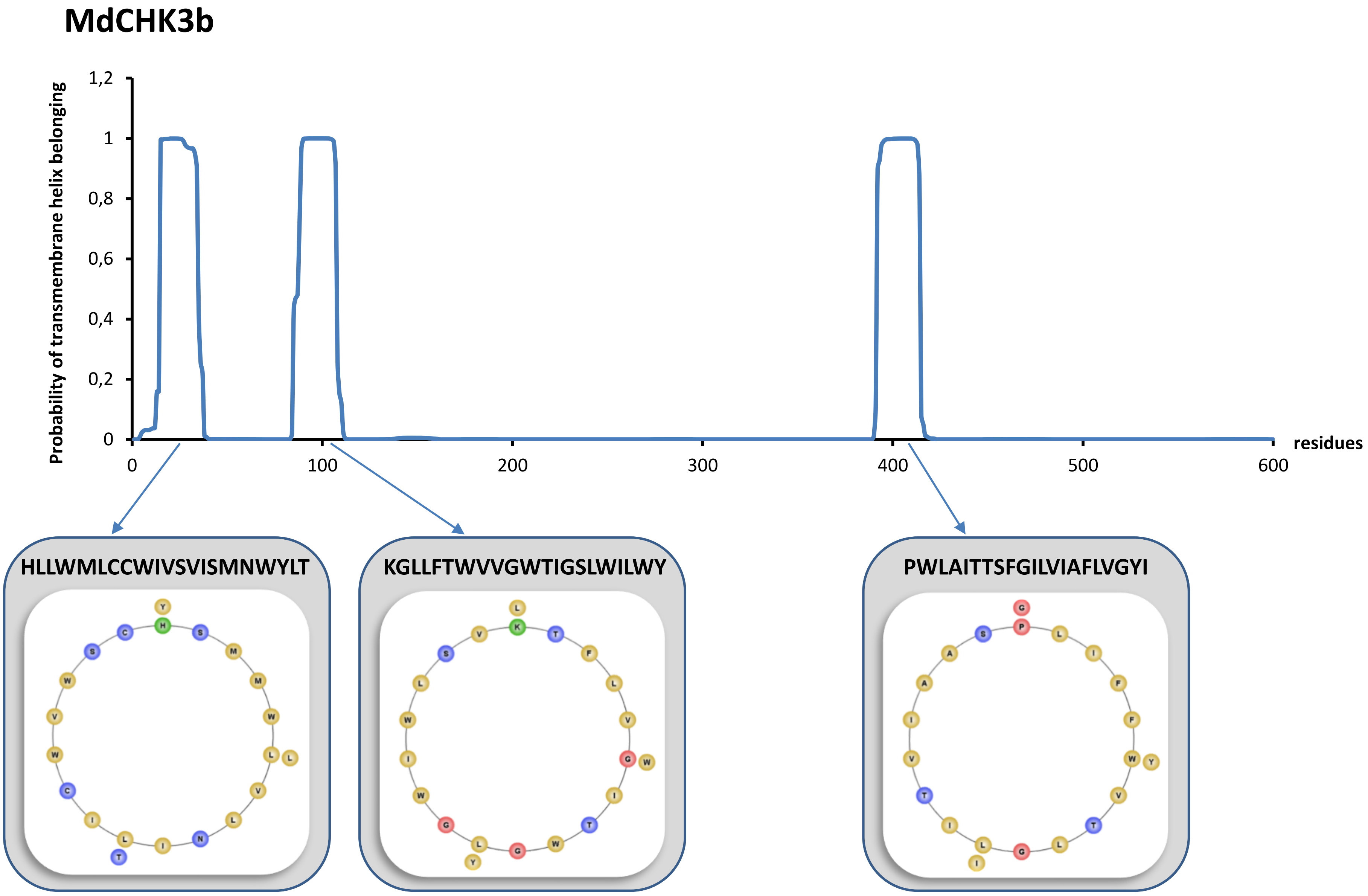

MdCHK3b
Probability of transmembrane helix belonging
1,2
1
0,8
0,6
0,4
0,2
0
0
100
200
300
400
500
600
residues
HLLWMLCCWIVSVISMNWYLT
KGLLFTWVVGWTIGSLWILWY
PWLAITTSFGILVIAFLVGYI

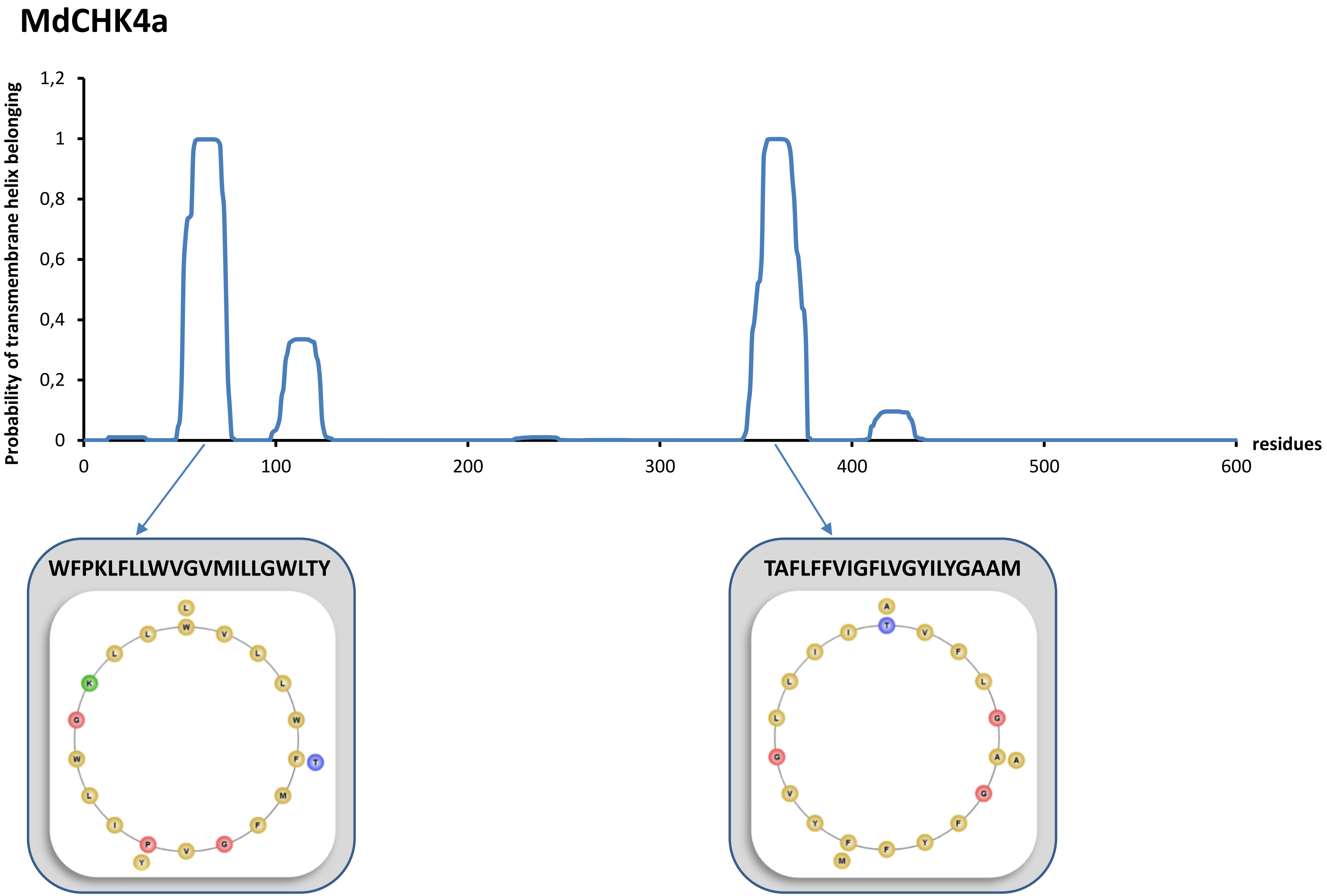

MdCHK4a
Probability of transmembrane helix belonging
1,2
1
0,8
0,6
0,4
0,2
0
0
100
200
300
400
500
600
residues
WFPKLFLLWVGVMILLGWLTY
TAFLFFVIGFLVGYILYGAAM

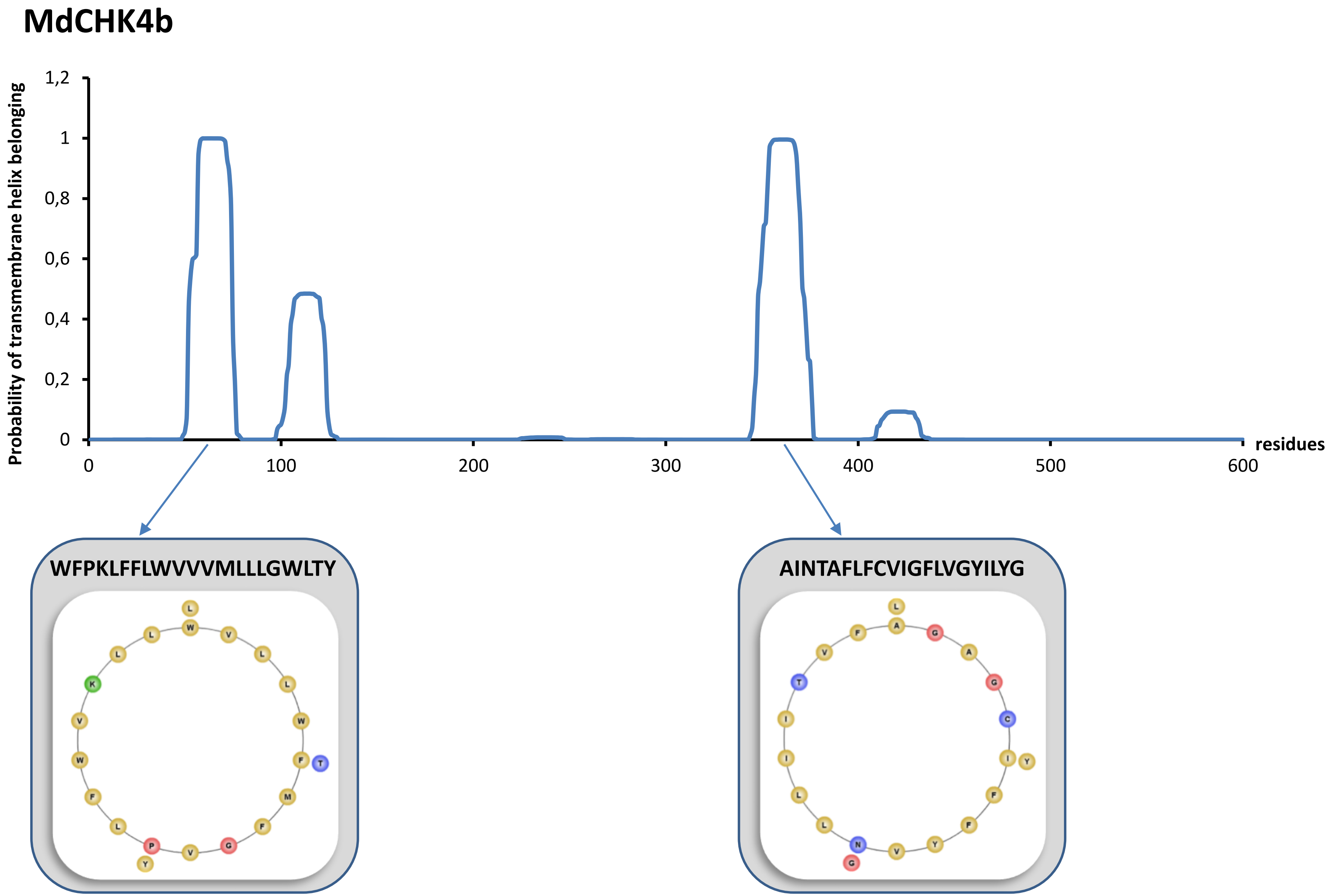


**Supplementary Figure S3.** Detection of putative transmembrane helixes at the N-terminal end of MdCHK. Probability of a residue to belong to a transmembrane helix as calculated for the 100-first amino acids of each MdCHK with a Markov model by the TMHMM server. Projection of the helical wheel has been done using http://www-nmr.cabm.rutgers.edu/bioinformatics/Proteomic_tools/Helical_wheel/

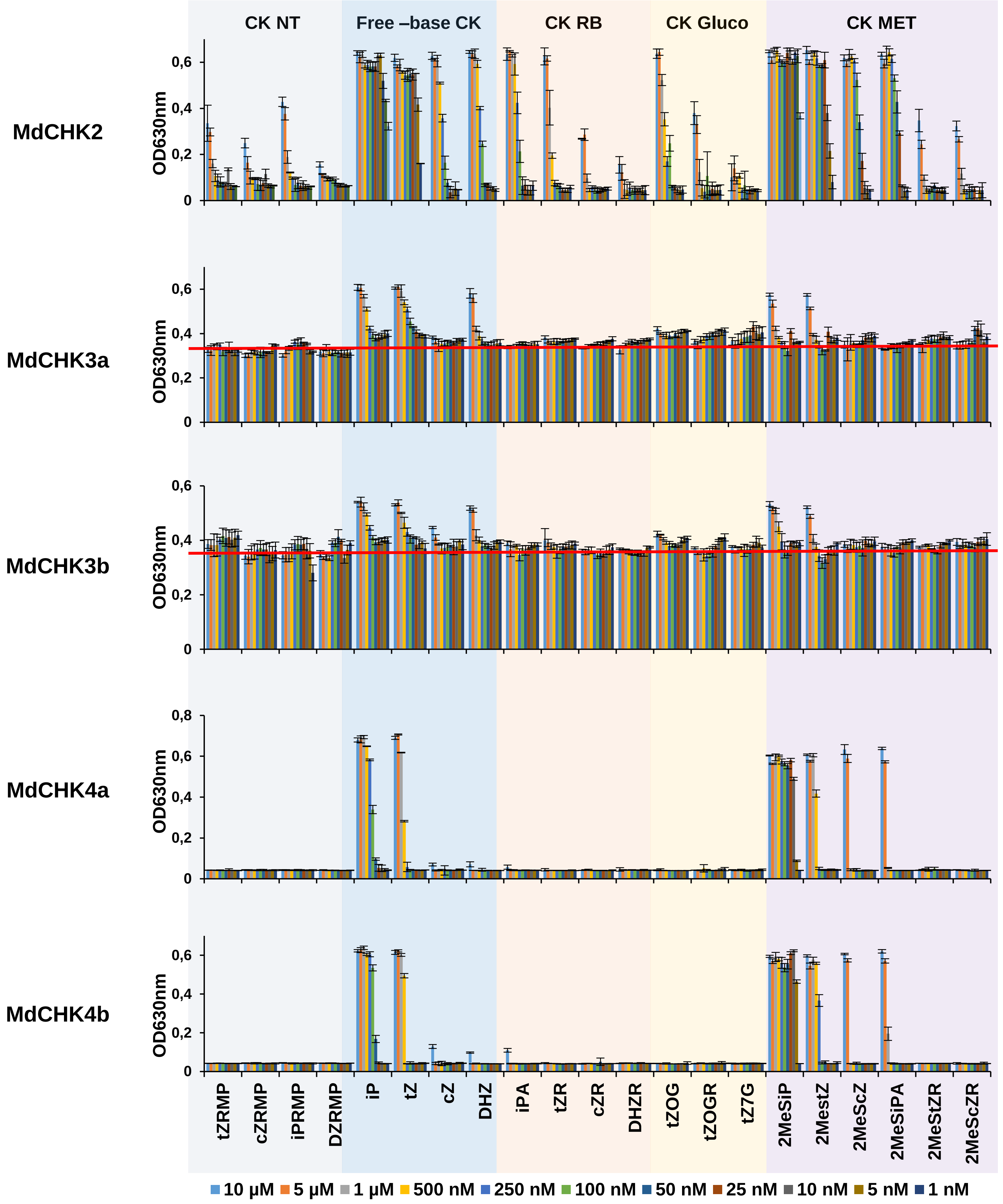


**Supplementary Figure S4. MdCHK receptors specificity and sensitivity towards cytokinins.** MdCHK-complemented *sln1* yeast were grown in presence of various cytokinins with different concentrations. The growth was measured at OD630nm after 48 hours. Basal growth of MdCHK3a and MdCHK3b is indicated by a horizontal red line. NT, nucleotide; RB, riboside; Gluco, glucoside; MET, methylthio; iPRMP, isopentenyladenosine 5'-monophosphate; *t*ZRMP, *trans*-zeatin riboside 5'-monophosphate; cZRMP, *cis*-zeatin riboside 5'-monophosphate; iP, isopentenyladenine; *t*Z, *trans*-zeatin; cZ, *cis*-zeatin; DHZ, dihydrozeatin; iPA, isopentenyladenosine; *t*ZR, *trans*-zeatin riboside; *c*ZR, *cis*-zeatin riboside; DHZR, dihydrozeatin riboside; DHZ, dihydrozeatin riboside 5'-monophosphate; *t*ZOG, *trans*-zeatin O-glucoside; *t*ZOGR; *trans*-zeatin O-glucoside riboside; *t*Z7G, *trans*-zeatin N7-glucoside; 2MeSiP, 2-methylthio-isopentyladenine; 2MeStZ, 2-methylthio-*trans*-zeatin; 2MeScZ, 2-methylthio-*cis*-zeatin; 2MeSiPA, 2-methylthio-isopentyladenosine; 2MeStZR, 2-methylthio-*trans*-zeatin riboside; 2MeScZR, 2-methylthio-*cis*-zeatin riboside.

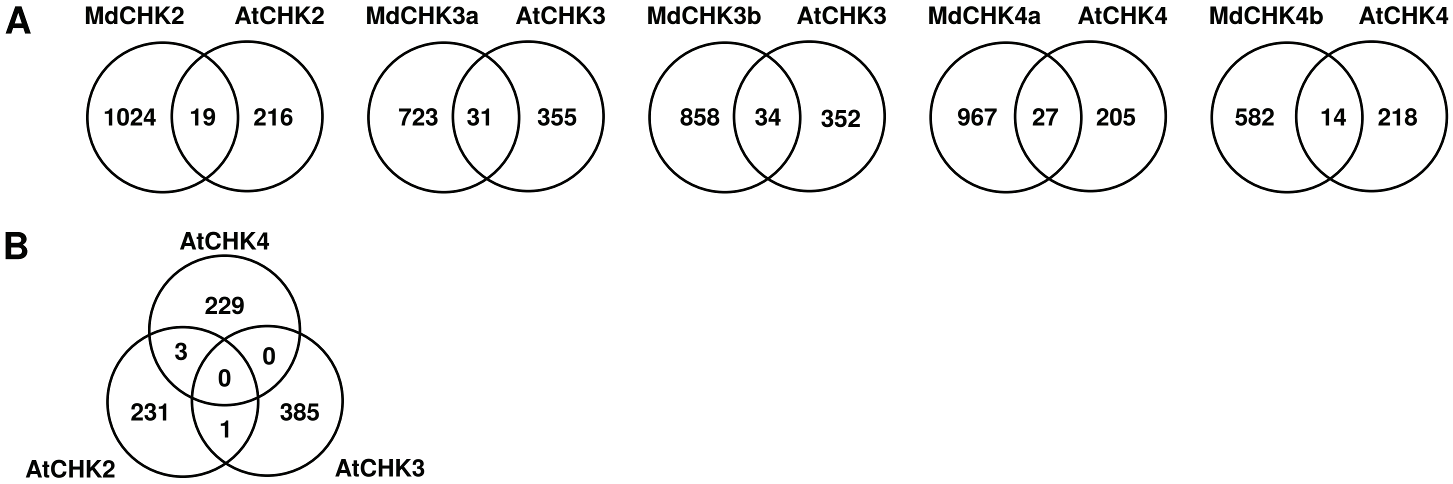


**Supplementary Figure S5.** CHK best co-expressed genes in Arabidopsis. A, lists of best co-expressed genes with each AtCHK were compared to their respective orthologs in apple tree. Orthology was obtained from Plaza 3.0. Venn diagrams depict the number of genes shared between CHK orthologs or specifically associated to them. B, lists of best co-expressed genes with each AtCHK were compared to display the poor conservation of functions associated among them.

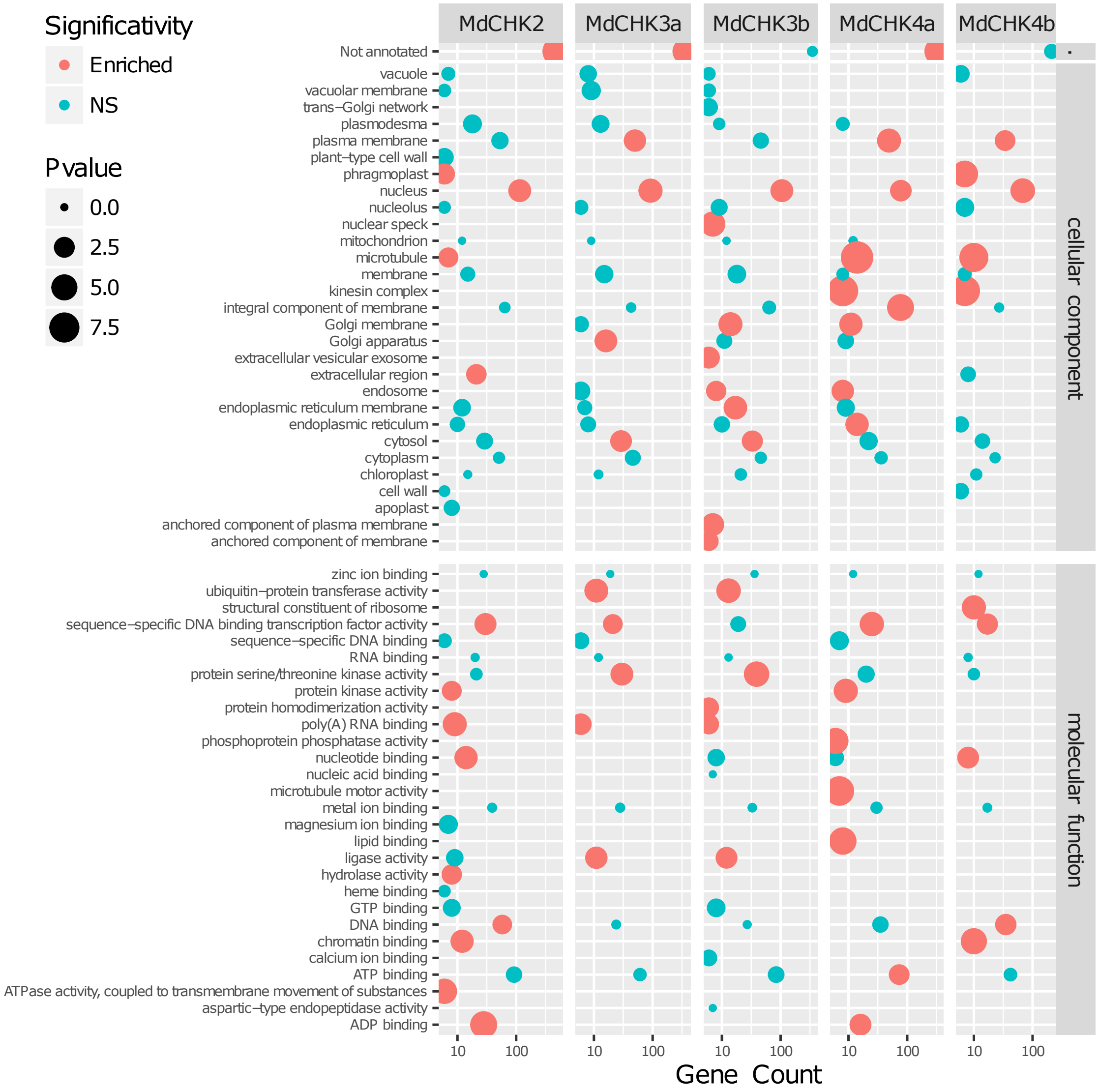


**Supplementary Figure S6.** Functional annotation of best co-expressed genes (HRR<500) with Gene Ontology (GO) terms. Only GO terms from « Cellular component » and « Molecular function » represented by 5 or more genes were kept. P-values correspond to GO term enrichment tests which were performed by comparison to hypergeometrical distribution.

**Supplementary Table S1**. List of primers used in the study.

| Primers | Sequence (5'→ 3') | Application |
|---|---|---|
| MdCHK2-F | ATGGAGACTTTGCATGGGACTAGC | *Cloning* |
| MdCHK2-R | TTAACCAGACTCAAAGAAACGTGC | |
| MdCHK3a-F | ATGAGTTTATTCCATGTATTTGGG | |
| MdCHK3a-R | TACGCGTTTCCAGTTGTAGTAGA | |
| MdCHK3b-F | ATGAGTGTTTTCCATGTATTTGGG | |
| MdCHK3b-R | TCACTCCAAGCTCCCAGTTGTTGT | |
| MdCHK4a-F | ATGGGTAAAGAGGAAACAAGAAGG | |
| MdCHK4a-R | TTAAGTTTTGAAGAACTTGGCC | |
| MdCHK4b-F | ATGAGCAAAGAGGAAACAAGAAGA | |
| MdCHK4b-R | TTAAGTTTTGAAGAACTTGGCA | |
| MdCHK2-SpeI-F | CTGAGAACTAGTATGGAGACTTTGCATGGGACTAGC | *Localisation BiFC* |
| MdCHK2-SpeI-R | CTGAGAACTAGTACCAGACTCAAAGAAACGTGC | |
| MdCHK3a-BamHI-F | CTGAGAGGATCCATGAGTTTATTCCATGTATTTGGG | |
| MdCHK3a-SpeI-R | CTGAGAACTAGTCGCGTTTCCAGTTGTAGTAGA | |
| MdCHK3b-SpeI-F | CTGAGAACTAGTATGAGTGTTTTCCATGTATTTGGG | |
| MdCHK3b-SpeI-R | CTGAGAACTAGTTCACTCCAAGCTCCCAGTTGTTGT | |
| MdCHK4a-SpeI-F | CTGAGAACTAGTATGAGCAAAGAGGAAACAAGAAGA | |
| MdCHK4a-SpeI-R | CTGAGAACTAGTAGTTTTGAAGAACTTGGCA | |
| MdCHK4b-SpeI-F | CTGAGAACTAGTATGGGTAAAGAGGAAACAAGAAGG | |
| MdCHK4b-SpeI-R | CTGAGAACTAGTAGTTTTGAAGAACTTGGCC | |
| MdCHK2-NotI-F | CTGAGAGCGGCCGCATGGAGACTTTGCATGGGACTAGC | *Yeast expression* |
| MdCHK2-NotI-R | CTGAGAGCGGCCGCTTAACCAGACTCAAAGAAACGTGC | |
| MdCHK3a-NotI-F | CTGAGAGCGGCCGCATGAGTTTATTCCATGTATTTGGG | |
| MdCHK3a-NotI-R | CTGAGAGCGGCCGCCTACGCGTTTCCAGTTGTAGTAGA | |
| MdCHK3b-NotI-F | CTGAGAGCGGCCGCATGAGTGTTTTCCATGTATTTGGG | |
| MdCHK3b-NotI-R | CTGAGAGCGGCCGCTCACTCCAAGCTCCCAGTTGTTGT | |
| MdCHK4a-NotI-F | CTGAGAGCGGCCGCATGGGTAAAGAGGAAACAAGAAGG | |
| MdCHK4a-NotI-R | CTGAGAGCGGCCGCTTAAGTTTTGAAGAACTTGGCC | |
| MdCHK4b-NotI-F | CTGAGAGCGGCCGCATGAGCAAAGAGGAAACAAGAAGA | |
| MdCHK4b-NotI-R | CTGAGAGCGGCCGCTTAAGTTTTGAAGAACTTGGCA | |
| qMdCHK2-F | AGTGGAGGCAGACTATCGTGA | *qPCR* |
| qMdCHK2-R | GCGTAGTCCTGTTGGTTTGGA | |
| qMdCHK3a-F | CTACAACTGGAAACGCGTAGTG | |
| qMdCHK3a-R | GCGATGATTTCATCAAATTTTC | |
| qMdCHK3b-F | CAGCATGGAGAAGTTTCTGCAG | |
| qMdCHK3b-R | TCACTCCAAGCTCCCAGTTGTTGT | |
| qMdCHK4a-F | GCCAAGTTCTTCAAAACTTAA | |
| qMdCHK4a-R | TAGTTCTAGGAGCAACTCTC | |
| qMdCHK4b-F | GCCAAGTTCTTCAAAACTTAA | |
| qMdCHK4b-R | CTAGGACTTTTAGGATCAAC | |
